# Epigenetic Risk Factors in PTSD and Depression

**DOI:** 10.3389/fpsyt.2013.00080

**Published:** 2013-08-07

**Authors:** Florian Joachim Raabe, Dietmar Spengler

**Affiliations:** ^1^Molecular Neuroendocrinology, Max Planck Institute of Psychiatry, Munich, Germany

**Keywords:** PTDS, depression, early-life stress, HPA axis, epigenetic programing, epigenetic variation

## Abstract

Epidemiological and clinical studies have shown that children exposed to adverse experiences are at increased risk for the development of depression, anxiety disorders, and posttraumatic stress disorder (PTSD). A history of child abuse and maltreatment increases the likelihood of being subsequently exposed to traumatic events or of developing PTSD as an adult. The brain is highly plastic during early life and encodes acquired information into lasting memories that normally subserve adaptation. Translational studies in rodents showed that enduring sensitization of neuronal and neuroendocrine circuits in response to early life adversity are likely risk factors of life time vulnerability to stress. Hereby, the hypothalamic-pituitary-adrenal (HPA) axis integrates cognitive, behavioral, and emotional responses to early-life stress and can be epigenetically programed during sensitive windows of development. Epigenetic mechanisms, comprising reciprocal regulation of chromatin structure and DNA methylation, are important to establish and maintain sustained, yet potentially reversible, changes in gene transcription. The relevance of these findings for the development of PTSD requires further studies in humans where experience-dependent epigenetic programing can additionally depend on genetic variation in the underlying substrates which may protect from or advance disease development. Overall, identification of early-life stress-associated epigenetic risk markers informing on previous stress history can help to advance early diagnosis, personalized prevention, and timely therapeutic interventions, thus reducing long-term social and health costs.

## Introduction

**Figure 1:**
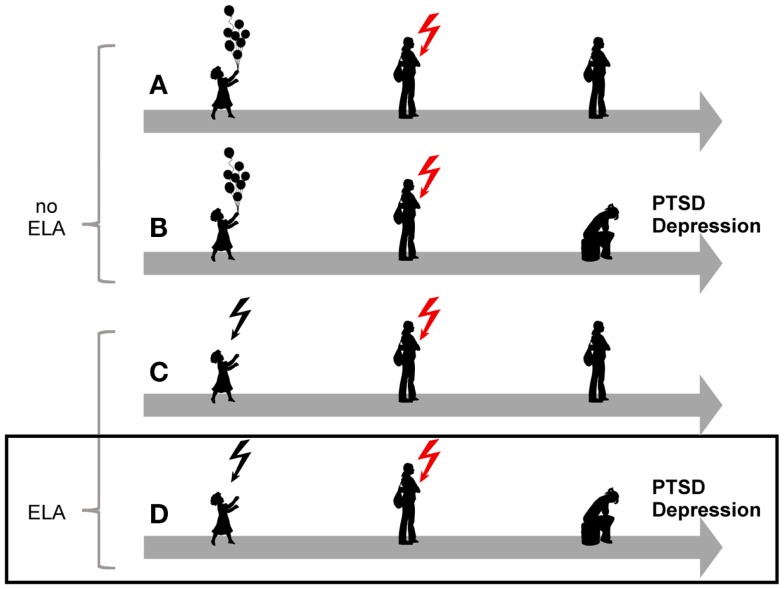
**Model for the role of early-life adversity for PTSD and depression**. In the absence of a history of early life adversity (ELA), adults can be resilient to disease upon exposure to severe trauma and stress **(A)** while others will develop PTSD or depression **(B)**. Similarly, not all children exposed to early life adversity will develop disease upon anew exposure to trauma and stress **(C)**. On the other hand, exposure to early life adversity in childhood can give rise to a vulnerable phenotype predisposing to disease upon anew exposure to trauma and stress**(D)**.

The overall burden of mental disorders – individual, societal, and economic – has been increasing in recent decades (1) and is greater than 10 years ago despite the availability of reasonably effective pharmacological and psychological interventions (2). Among mental disease posttraumatic stress disorder (PTSD) is a debilitating stress-related disease with prevalence rates amounting to 8%, and considerable higher rates among those living in high-violence areas and combat veterans (3–5). Originally thought of as a normative response to trauma, epidemiological studies showed that exposed subjects differed with respect to their later on risk for developing PTSD (6). This finding led to intense investigations to identify genetic and environmental factors associated with the onset, course, and treatment response of this disease. Overall, epidemiological and clinical studies have provided compelling evidence for a strong association between various forms of early life adversity, depression, and PTSD (Figure 1).

For this reason, we will refer the reader to the role of early life adversity for the development of depression whenever corresponding findings in PTSD are not available. This applies in particular to the role of experience-dependent epigenetic programing which is increasingly recognized as a mechanism in depression (7, 8). Moreover, given the central role of the hypothalamic-pituitary-adrenal (HPA) stress axis in response to early life adversity we will focus on its deregulation in both depression and PTSD. While PTSD and depression represent distinct entities, such a comparative approach can provide a conceptual framework for future studies in PTSD.

According to the National Comorbidity Survey Replication (9) early life adversity comprises interpersonal loss (parental death, parental divorce, and other separation from parents or caregivers), parental maladjustment (mental illness, substance abuse, criminality, and violence), maltreatment (physical abuse, sexual abuse, and neglect), life-threatening childhood physical illness in the respondent, and extreme childhood family economic adversity.

A landmark survey conducted at the Center for Disease Control showed a strong dose-response relationship between childhood adversities and mental health problems in adulthood (10). Patients suffering from major depression showed a fourfold increased risk for depression following multiple adverse exposures (11), a dose-response relationship between the severity of experienced childhood adversities and lifetime recurrent depression (12) and a twofold to fivefold increase in attempted suicide in up-growing children, adolescents, or later adults (13). These data corroborate with other representative surveys such as the National Comorbidity Survey (14), the Ontario Health Survey (15, 16), and the New Zealand Community Survey (17).

Similarly, individuals who were exposed to early life adversity are also more likely to develop PTSD (18, 19), to face re-exposure to trauma in adulthood, and to suffer from PTSD following trauma in adulthood (20–22) (Figure 1).

Hence, early life adversity is a powerful risk factor for mental diseases such as depression and PTSD and can predict a prolonged course and poorer response to treatment. The high incidence of child maltreatment represents an epidemic health problem and the long-term consequences of such trauma place a heavy burden on the healthcare system and society (23). According to the National Center of Child Abuse and Neglect approximately 3.4 million referrals, comprising alleged maltreatment of approximately 6.2 million children, were received across the US in 2011 (24). One-fifth of these children were found to be victims with disposition of substantiated (18.5%), indicated (1.0%), and alternative response victim (0.5%). All in all 676,569 victims of child abuse and neglect give rise to a unique victim rate of 9.1 victims per 1,000 children in the US population:

- more than 75% (78.5%) suffered neglect
- more than 15% (17.6%) suffered physical abuse
- less than 10% (9.1%) suffered sexual abuse
- more than four children (1,570 fatalities) died daily

These numbers recapitulated by and large prior findings (10, 25–28). Despite this compelling evidence for a link between early neglect, abuse, and later psychopathology (9, 29) knowledge about the molecular mechanisms underlying the long-term mental health consequences remains poor (30).

## The Vulnerable Brain

Early brain development is a time of great opportunities and great vulnerabilities. The architecture of the developing brain is constructed through an ongoing process that begins before birth and extends into adulthood. Brain development and architecture are built from the bottom up with simple structures providing the scaffold for the formation of more advanced structures over time. Research in the field of neuroscience has provided compelling evidence for the high plasticity of the developing brain as a function of experience which allows encoding of acquired information into lasting memories that normally subserve adaptation (31, 32). Sensitive periods refer to time windows during which developmental cues induce lasting programmable and organizational effects on neuronal substrates. Early adverse experiences in rodents and higher primates such as prenatal maternal stress, maternal separation, variable foraging demand, or naturally occurring low maternal care can lead to structural and functional changes in a connected network of brain regions implicated in neuroendocrine control, autonomic regulation, and vigilance (33–36). These neural changes converge on lifelong increased physiological and behavioral responses to subsequent stress (37). Ultimately, the enduring effects of early life adversity on the brain and its regulatory outflow systems, comprising the autonomic, endocrine, and immune systems, may lead to the development of a vulnerable phenotype with increased sensitivity to stress and risk for a range of somatic (38–40) and behavioral disorders (i.e., depression and PTSD) (9).

## The Central Role of the HPA Axis

**Figure 2:**
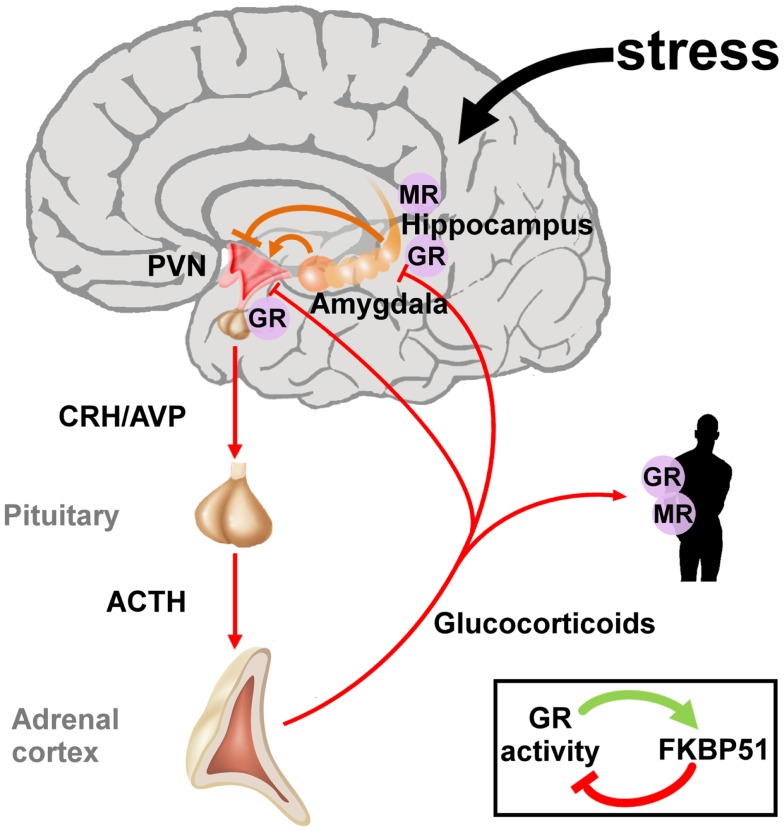
**The hypothalamic-pituitary-adrenal axis integrates and mediates the stress response to early life and later on adversity**. The perception of real and/or presumed physical and social threats causes activation of the hypothalamic-pituitary-adrenal axis. Anxious states arise from activation of the amygdala and magnify the stress response via neuronal projections to the paraventricular nucleus (PVN). The hippocampus plays an important role in the assessment of stressors and as a site of glucocorticoid receptor (GR) mediated negative feedback regulation. Release of the hypothalamic neuropeptides corticotrophin-releasing hormone (CRH) and arginine vasopressin (AVP) promotes the synthesis and secretion of adrenocorticotrophin (ACTH), a posttranslational cleavage product of anterior pituitary pro-opiomelanocortin mRNA (POMC). ACTH in turn stimulates the release of glucocorticoids from the adrenal glands. These hormones circulate throughout the whole body and the brain and bind to intracellular nuclear steroid receptors. Hippocampal mineralocorticoid (MR) receptors act to the onset of the stress response, while GR at the hippocampus, PVN, and anterior pituitary terminates the stress response. The GR further transactivates FKBP51 encoding a chaperon protein which curtails GR activity through a fast intracellular negative feedback loop.

Early life adversity can cause lasting structural and regulatory adaptations in the neuroendocrine system predisposing to or protecting from stress-related diseases later in life (41–43). Initial studies on PTSD focused on the HPA axis due to its central position in the neuroendocrine stress response (Figure 2). The two neuropeptides corticotrophin-releasing hormone (CRH) and arginine vasopressin (AVP) are secreted by the parvocellular neurons of the hypothalamic paraventricular nucleus (PVNh) and coordinate the behavioral and metabolic responses to stress. Binding to G-protein coupled receptors at the anterior pituitary gland induces co-operatively pro-opiomelanocortin (POMC) which is processed to adrenocorticotrophin (ACTH), opioid, and melanocortin peptides among others. Subsequently, ACTH stimulates the adrenal cortex to secrete cortisol (in humans) and corticosterone (in humans, rats, and mice). Corticosteroids activate ligand-gated glucocorticoid (GR) and mineralocorticoid (MR) receptors which are coexpressed in neurons of limbic structures. High levels of GR are additionally detected in the PVNh and the anterior pituitary. The MR is thought to serve in the assessment and onset of the stress response whereas GR, requiring higher amounts of glucocorticoids for activation, terminates the stress reaction (44).

In contrast to the PVNh, CRH neurons in the central nucleus of the amygdala project to the locus coeruleus and enhance the firing rate of its neurons resulting in increased noradrenaline release in the vast terminal fields of the ascending noradrenergic system (45). One of the principal noradrenergic targets are the CRH neurons of the PVNh, which respond to increased activity in the nucleus tractus solitarius, the dorsal medullary nucleus, and the locus coeruleus (46). Experimental lesions of these regions result in a significant decrease in stress-induced PVNh levels of noradrenaline, reduced plasma ACTH responses, and behavioral responses to stress (47, 48). In sum, CRH neurons in the PVNh and the central nucleus of the amygdala jointly mediate behavioral and endocrine responses to stress.

Although commonly adaptive, sustained activation of these stress circuits results in impairments. Elevated central CRH activity is associated with symptoms of anxiety and depression. This is most likely due to the CRH-induced stimulation of serotonergic and noradrenergic systems (49). Glucocorticoids and catecholamines enhance learning for information related to the stressor (50) whereas glucocorticoids impair attention and learning in relation to events not directly associated with the stressor (37). Additionally, glucocorticoids facilitate the behavioral effects of central Crh partly due to the upregulation of Crh in the central nucleus of the amygdala (51, 52).

## Long-Term Effects of Early-Life Adversity

In rodents and higher primates early-life stress lastingly influences the development of the central CRH systems underlying the expression of behavioral, emotional, autonomic, and endocrine responses to stress later in life (53, 54). Major depression frequently shows signs of a disinhibited HPA axis due to an increased parvocellular CRH expression which drives production and secretion of pituitary ACTH and subsequently cortisol (Table 1). CRH overexpression reflects an impaired negative GR feedback regulation as evidenced by a reduced inhibition of cortisol secretion after application of the synthetic glucocorticoid dexamethasone (55) (Table 1).

**Table 1:** **Neuroendocrine status in depressed patients without and with a history of early life adversity**.

	MD	Reference	MD + ELA	Reference
CRH	↑	Nemeroff et al. (139)	↑	Carpenter et al. (140)
	↑	Raadsheer et al. (141)		
	↑	Wang et al. (142)		
Cortisol	↑	Sachar et al. (143)	↓	Shea et al. (144)
	↑	Holsboer et al. (145)	↓	Heim et al. (146)
	↑	Holsboer et al. (147)	↑	Gerra et al. (148)
	↑	Wingenfeld et al. (149)	=	Heim et al. (150)
	↑	Carvalho Fernando et al. (151)	↑	Power et al. (152)
	↑	Hinkelmann et al. (153)		
	↑	Messerli-Bürgy et al. (154)		
	↑	Yilmaz et al. (155)		
DST	Cortisol ↑	Carroll et al. (156)	ACTH ↓cortisol ↓	Newport et al. (157)
	ACTH ↑ cortisol ↑	Modell et al. (158)		
	Cortisol ↑	Carvalho Fernando et al. (151)		
CST	ACTH ↑	Holsboer et al. (147)	ACTH ↓cortisol ↓	Heim et al. (146)
	ACTH ↓	Heim et al. (146)	Cortisol ↑	Heim et al. (150)

*MD, major depression; ELA, early life adversity; DST, dexamethasone suppression test; CST, corticotrophin-releasing hormone stimulation test; ACTH, adrenocorticotrophin hormone*.

Notably, depressed patients with a history of early child adversity were shown to differ in their neuroendocrine regulation. Although they shared signs of a hyperactive HPA axis, plasma cortisol is unaltered or reduced in part of these patients (Table 1). Moreover, their response to psychotherapy alone appeared superior to antidepressant therapy when compared to patients without a history of early child adversity (56).

Adult PTSD patients show a distinct neuroendocrine profile characterized by centrally elevated CRH, low plasma cortisol, and enhanced suppression of plasma cortisol and ACTH following the dexamethasone suppression test (Table 2). An increased responsiveness of both peripheral and central GR has been suggested as a plausible cause for hypocortisolemia (Table 2).

**Table 2:** **Neuroendocrine status in PTSD patients without and with a history of early life adversity and in healthy controls exposed to early life adversity**.

	PTSD	Reference	ELA	Reference
CRH	↑	Bremner et al. (159)	↑	Lee et al. (160)
	↑	Baker et al. (161)		
	↑	de Kloet et al. (162)		
Cortisol	↑ → ↓ *Ch*	Pervanidou et al. (60)	↓ *Ch*	Carlson and Earls (163)
	↓ *ELA*	Bremner et al. (164)	↓ *Ch*	King et al. (165)
	↓ *ELA*	Brand et al. (166)	↓ *Ch*	Kliewer (167)
	↓	Yehuda et al. (168)	↓ *Ch*	Bevans et al. (169)
	↓	Boscarino (170)	↓ *Ch*	Badanes et al. (171)
	↓	Yehuda et al. (172)	↓ *Ch*	Hauser et al. (173)
	↓	Thaller et al. (174)	≈*Ch*	Bruce et al. (57)
	↓	Rohleder et al. (175)	↑ *Ch* → ↓	Trickett et al. (59)
	↓	Bierer et al. (176)	↓	Resnick et al. (61)
	↓	Brand et al. (177)	↓	Brewer-Smyth and Burgess (178)
	↓	Wingenfeld et al. (149)	↓	Flory et al. (179)
	↓ *RF*	Resnick et al. (61)	↓	Power et al. (152)
	↓ *RF*	McFarlane et al. (62)	≈*Ad*	Weissbecker et al. (180)
	↓ *RF*	Delahanty et al. (63)	≈*Ad*	van der Vegt et al. (181)
GR-r	↑	Yehuda et al. (182)	↑	Heim et al. (146)
	↑	Yehuda et al. (183)		
	↑	Yehuda et al. (184)		
DST	Cortisol ↓	Yehuda et al. (183)	Cortisol ↓ *Ch*	Goenjian et al. (58)
	ACTH ↓ *ELA*	Duval et al. (185)	Cortisol ↓	Stein et al. (186)
	ACTH ↓ cortisol ↓	Yehuda et al. (187)	Cortisol ↓	Heim et al. (188)
	ACTH ↓	Ströhle et al. (189)		
CST	ACTH ↓ cortisol ↓	Ströhle et al. (189)	Cortisol ↑	Heim et al. (188)
			Cortisol ↓	Carpenter et al. (190)
			ACTH ↑	Heim et al. (146)

*PTSD, posttraumatic stress disorder; ELA, early life adversity; GR-r, glucocorticoid receptor reactivity; DST, dexamethasone suppression test; CST, corticotrophin-releasing hormone stimulation test; ACTH, adrenocorticotrophin hormone. Indices are; RF, risk factor for PTSD; Ch, children; ≈Ch or ≈Ad, flattened diurnal cortisol in children or adults*.

While hypocortisolemia in depressed patients is confined to those with a history of early life adversity (Table 1, right), PTSD patients are affected in either case (Table 2, left). This raises the question of whether hypocortisolemia precedes trauma exposure or whether it is a long-term consequence thereof promoting the manifestation of either depression or PTSD.

In this regard, longitudinal studies have shed some light on the development of HPA-axis deregulation in traumatized children. From an early age on, maltreatment, neglect, physical, and sexual abuse manifest with low levels of cortisol which persist into adulthood (Table 2, right). Furthermore, abused children display flattened diurnal cortisol levels (57) and increased cortisol suppression after administration of dexamethasone (58). These results corroborate the concept of an enhanced GR response and negative feedback function. However, sexually abused girls initially showed higher levels of cortisol after trauma exposure. Hypercortisolemia decreased, however, during growing up and cortisol remained low in young adults when compared to their unabused peers (59). A similar cortisol pattern has been detected in pediatric patients who experienced motor vehicle accidents and developed PTSD at 6 months. Initially, they had high levels of cortisol which passed over into hypocortisolemia at 6 months (60). Taken together, these findings suggest that trauma exposure associates with an acute stress response followed by hypocortisolemia and GR hyperresponsiveness in vulnerable subjects. This raises the important question of whether hypocortisolism precedes clinical manifestation of PTSD and thus represents a potential marker for later on risk for illness (Table 2). In this respect, longitudinal studies identified low levels of cortisol as a risk factor to develop PTSD after trauma exposure (61–63).

In conclusion, neuroendocrine findings in PTSD and individuals exposed to early life adversity indicate that hypocortisolemia *per se* does not result in PTSD but seems to increase the risk to develop disease following additional trauma exposure. The cellular and molecular mechanisms causing hypocortisolemia due to enduring deregulation of the HPA axis and how they interact with later on trauma to manifest as PTSD are presently largely unknown.

In the following parts, we will explore the possibility of epigenetic mechanisms to encode traumatic experiences. We will further argue that insight from experience-dependent epigenetic programing by early-life adversity in depression can serve as guidance to advance translational studies in PTSD.

## A Guide to DNA Methylation

The epigenetic landscape has unfolded at multiple layers involving DNA methylation, histone modifications, non-coding RNA, and nucleosome positioning; along with DNA sequence. For the purpose of this review we will consider only the former two and refer the interested reader to recent reviews (64–66).

As a stable, though potentially reversible, repressive mark, DNA methylation is considered to represent an important player in epigenetic control of transcription (67). DNA methylation is carried out by a family of DNA methyltransferases (DNMTs) comprising DNMT1, DNMT3A, DNMT3B, and DNMT3L. DNMT1 primarily methylates hemimethylated DNA *in vitro* and is recruited to replication foci during S phase, while DNMT3A and DNMT3B preferentially recognize unmethylated CpG dinucleotides and regulate *de novo* methylation during development.

**Figure 3:**
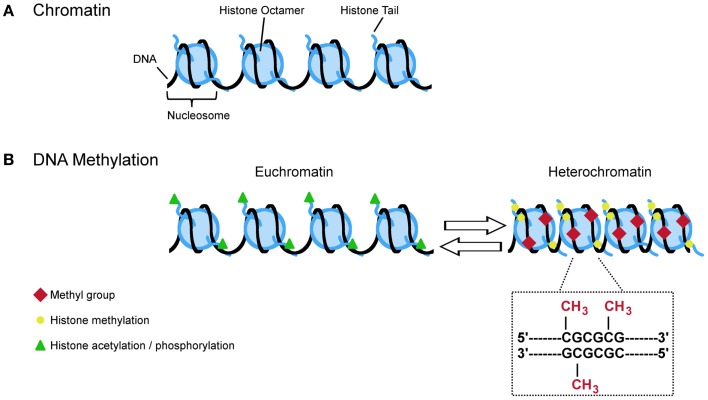
**A (simplified) guide to DNA methylation**. **(A)** Approximate 147 bp of DNA are wrapped around the nucleosome which is made up by the core histones H2A, H2B, H3, and H4. The free ends of the histone tails serve as substrate for posttranslational modifications (e.g., acetylation, phosphorylation, and methylation) that influence the configuration of chromatin. Opened states (euchromatin) allow transcription while closed states (heterochromatin) restrict the transcriptional machinery. **(B)** Euchromatin is characterized by acetylation and phosphorylation (green triangles) whereas methylation (yellow circles) is more often found at heterochromatin. Specific histone modifications and DNA methylation reciprocally influence each other in deposition. For example, histone methylation at H3K9, H3K27, and H4K20 promotes DNA methylation at CpG dinucleotides. This covalent modification refers to the transfer of methyl group to cytosine resides at gene regulatory regions. Hypermethylation typically links to lasting transcriptional repression while hypomethylation favors gene expression.

Specific histone modifications and DNA methylation reciprocally influence each other in deposition (Figure 3). Mechanistically, components of the histone methylation system associate physically with one or more DNMTs. For example, trimethylation of H3K9, H3K27, and H4K20 emerged as a prerequisite for subsequent DNA methylation. The methyltransferases SUV39H1/2 and EZH2 catalyzing H3K9 and H3K27 methylation, respectively, bind directly to DNMT1, DNMT3A, or DNMT3B and facilitate their recruitment to target loci (68, 69). Similarly, the recruitment of HP1 (heterochromatin protein 1) to constitutive heterochromatin results from SUV39H1/2-mediated trimethylation of H3K9 while euchromatin binding depends on dimethylation catalyzed by the histone methyltransferase G9A (70). Once bound, HP1 can directly interact with DNMT3A to guide DNA methylation (71). Conversely, histone methylation can also interfere with DNA methylation. DNMT3L, lacking catalytic activity, specifically recognizes the extreme amino terminus of the core histone H3 in a methylation sensitive manner. Methylation of H3K4 but not of other residues blocks interaction with DNMT3L and the subsequent recruitment of DNMT3A2 (72).

Taken together, histone methylation can both facilitate and impair the recruitment of DNMTs and intermediary factors in a context dependent manner to impose changes in DNA methylation and the long-term control of gene activity (Figure 3).

The globally methylated, CpG poor genomic landscape is punctuated by CpG islands (CGIs). Approximately 70% of all annotated gene promoters are associated with a CGI that are typically free of DNA methylation (73). In mammals tissue- and cell type-specific DNA methylation is detected in a small percentage of 5′ CGI promoters whereas most DNA methylation occurs in intra- or inter-genic regions which unexpectedly correlates with increased rather than decreased transcription (74–76) or alternatively with elective promoter usage (77).

Active DNA demethylation in mammals remains presently subject of intense investigation as cytosine deamination, oxidation, and base excision repair enzymes have been suggested in a variety of combinations (78).

A second, more recently discovered mechanism implicates the actions of ten eleven translocation (TET) proteins which convert 5-methylcytosine to 5-hydroxymethylcytosine (5-hmC) by an oxygen-, and α-ketoglutarate-dependent mechanism, then to 5-formylcytosine and hereafter to 5-carboxycytosine (79). This modified residue is excised by glycosylases to be replaced by cytosine via the base repair system (80). Interestingly, hydroxymethylation can be selectively recognized by DNA-binding proteins raising the prospect that it serves a biological function on its own (81). For example, Mbd3 preferentially binds to 5-hmC relative to 5-methylcytosine to confer gene repression (82).

In sum, DNA methylation occurs at different gene regulatory regions controlling transcription, alternative promoter usage, and splicing. Methylation of CpG residues is a stable though potentially reversible mark catalyzed by enzymatic conversion.

## An Epigenetic Archive

The “DNA ticketing theory of memory” was proposed some 40 years ago by Griffith and Mahler to account for the extraordinary persistence of memory (83). Given that “nerve cells do not normally divide in adult life and their DNA is generally considered to be metabolically stable” they hypothesized that “the physical basis of memory could lie in the enzymatic modification of the DNA of nerve cells” whereby “the modification consists of methylation (or demethylation).” This concept has been reviewed by Holliday who proposed that “the initial signal which is to be memorized, switches the DNA from a modified to an unmodified state, or vice versa;” thereby, “demethylation is a more likely possibility since from existing evidence this may activate a gene” (84). This hypothesis has been largely ignored because DNA methylation was originally thought of as part of a stable, epigenetic cellular memory system that controls gene silencing via chromatin structure. Hence, postmitotic cells were considered to be refractory to any changes in DNA methylation. Recent reports suggest, however, that DNA methylation is also involved in controlling the dynamics and plasticity of gene regulation, particularly during differentiation (85). Moreover, DNA methylation in postmitotic neurons can respond to social experiences and encode lasting changes in gene expression (86). In this regard, experience-dependent DNA memories can be described as the sequential process of a social and/or physical event registered by sensory and cognitive brain areas. The subsequent activation of intracellular signaling pathways, coupled to the epigenetic machinery, and ultimately its recruitment to specific gene loci, enables to erase, establish, and maintain epigenetic marks. Admittedly, not all examples of early-life stress-dependent changes in DNA methylation can satisfy these criteria (7).

For the purpose of this review, we will focus on those genes of the HPA axis known to be lastingly deregulated in both depression and PTSD and ask whether epigenetic programing contributes to these processes. Experience-dependent programing of the HPA axis has been intensively studied in rodents and examples given below serve to illustrate the overarching concept without attempting to provide a comprehensive survey of this field. In considering epigenetic programing of key regulators of the HPA axis, a developmental perspective (i.e., embryonic, prenatal, postnatal, and adult stages) is adopted whenever possible to illustrate that the brain is susceptible to epigenetic programing across the entire life span.

## Experience-Dependent Programing of CRH

Parvocellular hypothalamic CRH-expressing neurons participate both in the peripheral and central stress systems by governing secretion of ACTH from the pituitary contributing to the peripheral, neuroendocrine stress response, and by modulating stress-related behavior including anxiety as well as learning and memory. The CRH peptide is additionally detected in neocortex and in limbic regions including the central nucleus of the amygdala, the dorsal and ventral part of the bed nucleus of the stria terminalis, and the hippocampus. Further sites comprise the locus coeruleus, dorsal and median raphe, periaqueductal gray, nucleus of the solitary tract, and cerebellar complex. For the purpose of this review, we will focus in the following section largely on stress-dependent programing of Crh in the PVNh.

Early-life experience can evoke lasting changes in Crh expression levels in the PVNh as evidenced by several paradigms comprising onetime or repeated separation from the mother for up to 24 h, manipulation of maternal behavior by “handling” and limiting nesting materials for the dam during the first weeks of life. In this respect, maternal separation for 24 h has been documented to either not influence (87) or reduce basal C*rh* gene expression in rat (88) and mice PVNh (89). Of note, prolonged maternal separation for 8 h leads to desensitization of the HPA axis with reduced corticosterone after replication of prolonged maternal separation (90, 91). In response to acute stress, maternal deprivation leads, however, to higher transcription of the C*rh* gene and rapid secretion of ACTH and glucocorticoids (87, 92–94).

Enhanced maternal care due to natural variation (95, 96) reduces C*rh* expression in the PVNh in the adult offspring, curtails hormonal responses to stress, and enhances hippocampal GR levels. Similarly, daily brief (15 min) separations from the mother during postnatal week 1 up to 3 promotes an adult phenotype characterized by a sensitization of the HPA axis with an attenuated stress response (33, 97) associated with reduced basal expression of Crh in the PVNh and increased hippocampal expression of the GR (98, 99). Hereby, the reduction of C*rh* expression in the PVNh precedes further changes at different levels of the HPA axis compatible with the idea that C*rh* mediates handling-evoked maternal care to adaptation of the HPA axis (99, 100).

Sustained early-life stress can also be generated by limiting the amount of nesting material which creates an enduring stressor for the dam. This leads to fragmented maternal care and imposes an additional stressor on the pups (101, 102). By the end of 1 week of postnatal stress (P9), Crh mRNA expression in the PVNh of the early-life stress group is significantly reduced compared to undisturbed controls (102). Reduced C*rh* expression may reflect altered glucocorticoid negative feedback and/or increased Crh release concomitant with a failure of acute stress-induced Crh production.

Collectively, these exemplary studies in rodents illustrate that early trauma and stress can cause sustained changes in Crh expression which critically depend on the developmental stage of the limbic-HPA axis. Are then any of these changes in C*rh* expression related to epigenetic mechanisms such as DNA methylation?

## Epigenetic Programing of C*rh*

**Figure 4:**
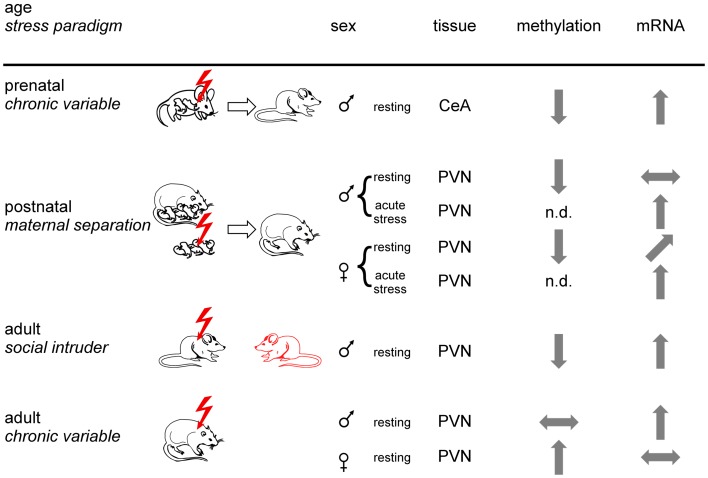
**Experience-dependent epigenetic programing of C*rh***. Prenatal, postnatal, and adult mice or rats were exposed to different stress paradigms (chronic variable, maternal separation, or social intruder stress). Sexes are indicated by respective symbols. Results from C*rh* promoter methylation and expression are depicted for the paraventricular nucleus (PVN) and the central amygdala (CeA). Tissues were isolated under resting conditions or following application of an acute stressor. Arrows show increased, decreased, and unaltered CpG methylation and Crh mRNA expression, respectively, for the indicated brain regions. Non-determined (n.d.) refers to the absence of information. In sum, epigenetic programing of C*rh* occurs in a stressor-, sex-, and tissue-specific manner.

The fetus grows up in the womb protected by the placental barrier which shields from any adversity that might arise in the maternal environment. Severe stressors and strains can, however, compromise the placental barrier and expose the developing fetal brain to maternally derived substances, such as cytokines or stress hormones. The latter can trigger increased vulnerability to depression, anxiety, schizophrenia, and autism (103). In this respect, male but not female mice offspring exposed to chronic variable stress early in gestation (embryonic day 1–7) displayed impaired behavioral stress responsivity, anhedonia, and an increased sensitivity to treatment with a selective serotonin reuptake inhibitor (104). Increased Crh expression in the amygdala is associated with decreased promoter methylation (Figure 4), while decreased GR expression in the dorsal hippocampus correlated with increased methylation at the NGFI-A binding site of exon 1_7_ (see below). Notably, the fetal brain was unformed at the time the stressor was applied to the mother indicating that sex-specific changes in fetal development and long-term adaptation of the HPA axis might be mediated through effects on the developing placenta. In this regard, male control placentas showed lower Dnmt1 expression compared to females. Moreover, increases in placental Dnmt1 in response to prenatal stress were largely confined to females. How increased Dnmt1 expression might counteract prenatal stress-dependent perturbations in placental gene expression remains presently unknown.

In a postnatal study, rats exposed to early-life stress (maternal separation) were studied for HPA-axis responses to acute restraint stress and C*rh* promoter methylation in the PVNh nucleus and the central nucleus of the amygdala (105). Despite slightly lower plasma ACTH, plasma corticosterone levels in female rats subjected to early-life stress were significantly higher in basal conditions and after exposure to acute restraint stress. In males, early-life stress did not affect basal plasma corticosterone levels, but in females the increases after restraint were significantly higher in early-life stressed males than in controls. In accord with these findings, Crh mRNA expression in the PVNh was increased under resting and acute stress conditions in females while increases in males occurred only under acute stress conditions (Figure 4). Moreover, Crh expression in the central amygdala was unaffected in either condition in both sexes. Early-life stress associated with hypomethylation of the C*rh* promoter in the PVNh in either sex and mapped to two CpG residues localized next to and at the dyad symmetry axis of a cyclic AMP responsive element (CRE). Methylation of the latter CpG residue impaired DNA binding of the activated form of the CRE binding protein (p-CREB) compatible with the idea that early-life stressed induced hypomethylation facilitates CREB binding and subsequently basal and activated Crh transcription.

Exposure to social defeat over 10 consecutive days imposes sustained social stress on adult male mice resulting in anhedonia and social avoidance. This well-known paradigm associated with demethylation at the proximal C*rh* promoter (106). As a result, Crh mRNA expression in the PVNh was significantly increased (Figure 4). Interestingly, Crh demethylation was confined to males displaying signs of stress while those being unaffected escaped demethylation and Crh overexpression. Given that genetically homogenous mice were housed and tested under standardized operating procedures this differential response suggests that subtle differences in the previous life histories can modify the outcome from exposure to stress. Application of imipramine over 3 weeks, a clinically relevant time period, improved the social avoidance behavior and reversed changes in C*rh* promoter methylation and gene expression. C*rh* demethylation was preceded by decreases in Dnmt3b and Hdac2 expression, while Gadd45b expression was temporarily increased. In this regard, Gadd45b has been shown to subserve demethylation by facilitating recruitment of the DNA repair machinery (107, 108).

In a further study, adult male and female rats were exposed to chronic variable stress after which C*rh* expression and promoter methylation was measured in the PVNh, the bed nucleus of the stria terminalis and the central amygdala (109). Chronic variable stress produced C*rh* promoter hypermethylation at specific CpG dinucleotides in all tissues examined in females while the effects in males were confined to the stria terminalis and the central amygdala. In the PVNh, chronic variable stress increased Crh expression in the males (in the absence of changes in methylation), while the Crh peptide decreased in females most likely due to increased promoter methylation (Figure 4). This study firstly evidenced sex-specific epigenetic programing of C*rh* and illustrates the importance of sex differences in brain epigenetics (110). The functional implications of decreased Crh expression in females in the presence of elevated corticosterone levels requires, however, further investigations.

**Figure 5:**
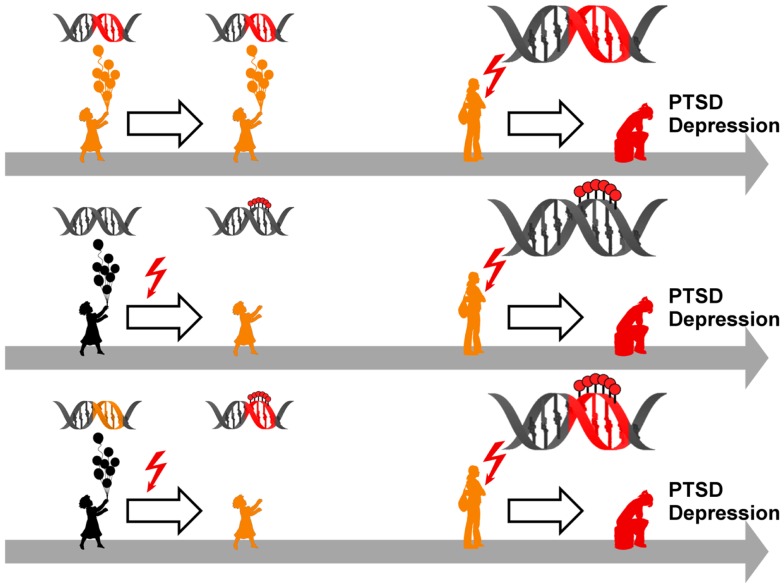
**Model for epigenetic risk factors in PTSD and depression**. Genetic mutations (red segment of the DNA double helix) can confer an increased vulnerability to PTSD and depression which manifest following anew exposure to stressful life events (red flash). Although carrying a predisposition to disease (symbolized by orange colored child and adolescent), such individuals can stay healthy in the absence of trauma and stress (upper panel). Early life adversity (red flash) can elicit epigenetic programing of stress genes via DNA (de-)methylation (symbolized by red lollipops on DNA double helix) leading to altered expression. These alterations confer an increased vulnerability to later on trauma and stress and ultimately result in manifestation of PTSD and depression (middle panel). Genetic mutations (orange segment of the DNA double helix) can serve as a substrate for epigenetic programing in response to early trauma and stress (red flash) via DNA (de-)methylation (symbolized by red lollipops on red segment of DNA double helix). This pre-activation can result in PTSD and depression following anew exposure to trauma and stress (lower panel).

Together, these studies show that epigenetic programing of *Crh* can occur in a stressor-, tissue-, and sex-specific manner. Moreover, differences in the previous life history might protect from epigenetic programing. Subsequent variations in DNA methylation at the C*rh* gene can serve to distinguish the transcriptional response to later stress-related stimuli. Experience-dependent programing of gene expression potential can thus confer an increased risk to anewed stress exposure and the manifestation of stress-related diseases such as depression and PTSD (Figure 5).

## Experience-Dependent Programing of Avp

Newborn rats exposed to repeated brief maternal separation and handling (PND 1–10) showed as adults reduced social investigative interactions and increased aggressive behavior with a higher frequency of attacks in the social interaction test. The latter behavior was detected only in males which displayed an increased number of Avp-positive magnocellular neurons in the PVN (111).

A related phenotype was detected following maternal separation in mice (112). While both sexes displayed increased anxiety-related behavior (reduced time spent on open arms in the elevated plus-maze and reduced exploration time of novel objects) this alteration associated with sex-specific differences in Avp expression. In this respect male but not female mice showed increases in Avp immunoreactivity in magnocellular neurons of the PVN and the lateral hypothalamus.

Sex differences in response to early-life adversity are not limited to Avp but extend to the receptor level. Adult rats that were reared by high licking and grooming mothers showed increased Avp V1a receptor binding in the central nucleus of the amygdala (113).

A recent study in human strengthens the importance of sex differences in AVP expression and suggests possible therapeutic consequences (114). PTSD is associated with problems in intimate relationships, partly due to impaired social cognition. Attention to partner expressions of anger was investigated as an indicator of distress and need for affiliative behaviors to repair the relationship bond. AVP administration enhanced men’s attentional engagement with their partners’ expressions of anger and alleviated the negative impact of PTSD on this social cognitive process. Moreover, men’s morning urinary AVP levels were negatively correlated with their PTSD severity. In contrast, no such effects were detected among women or for attention to unfamiliar men’s or women’s anger expressions.

Collectively, early-life adversity preferentially programs the expression of hypothalamic Avp and its receptor in male rodents. Moreover, deficits in social cognition in male PTSD patients are improved following AVP administration.

## Epigenetic Programing of *Avp*

Maternal separation (3 h per day from postnatal day P 1–10) has been used to study early-life stress in male mice. Consistent with previous studies, adult mice displayed a hyperactive HPA axis characterized by corticosterone hypersecretion under resting conditions and following application of an acute stressor. These findings associated with enhanced immobility in the forced swim test and memory deficits in an inhibitory avoidance task (115).

Whereas hypothalamic Crh mRNA expression was not affected by early-life stress, the paradigm resulted in a sustained upregulation of Avp. This response was confined to the PVNh coordinating the stress response whereas Avp expression in the supraoptic hypothalamic nucleus which controls fluid homeostasis remained unaffected. Increased Avp expression correlated with reduced levels of DNA methylation at multiple CpG residues at a downstream enhancer region which serves to fine-tune gene expression. Hypomethylation evolved gradually in response to maternal separation to reach a maximum at 6 weeks. Thereafter, changes in DNA methylation declined slowly but were still detectable after 1 year (115).

Hypomethylation localized to high affinity DNA-binding sites for the methyl-CpG-binding protein Mecp2. This epigenetic reader serves as a scaffold for the assembly of DNMTs and histone deacetylases which can rewrite epigenetic marks to induce gene silencing (86, 116). Hypomethylation in 6-week-old early-life stress treated mice reduced Mecp2 binding at the *Avp* enhancer and consequently promoted increased gene expression.

Early-life stress treated mice showed, however, no signs of hypomethylation directly after termination of the stressor (postnatal day 10) although Avp expression was robustly increased. This finding indicated that stress signals unrelated to DNA methylation appear to control binding of Mecp2 at earlier stages. In support of this concept, depolarization-dependent Ca^2+^ influx and activation of calmodulin kinases has been shown to cause Mecp2 phosphorylation. This event impairs Mecp2 DNA binding and derepresses target genes (115). In accord with this hypothesis, early-life stress treated mice showed increased Mecp2 phosphorylation in parvocellular Avp-expressing neurons at postnatal day 10.

Taken together, these results suggest a stepwise mode for epigenetic programing of *Avp*. Initially, early-life stress leads to phosphorylation of the epigenetic reader Mecp2. This modification favors dissociation from the *Avp* enhancer and promotes increased expression. Moreover, due the absence of Mecp2, epigenetic writers like DNMTs and histone deacetylases loose their docking sites and will fall short to maintain DNA methylation. As a result *Avp* hypomethylation will gradually evolve and leave a persistent memory trace of the early-stress event encoding an altered stress regulation.

This stepwise mode of epigenetic programing of Avp in response to early trauma involves the transition from stress-induced posttranslational modifications (so called soft-wiring) to stable DNA modifications (so called hard wiring). Although there are so far no related studies in humans, we want to point out that changes following from exposure to early life adversity appear initially reversible. They might, however, solidify over time or upon re-exposure to stress. In this respect, early interventions such as improvements in parenting and the social environment might delay or protect individuals exposed to early trauma to develop disease at later stages.

## Experience-Dependent Programing of GR

Experience-dependent programing of the HPA axis also applies to negative feedback regulators like the GR. Enhanced maternal care during the first 10 days of life (95) and daily short (15 min) periods of maternal separation during the first postnatal week (98, 99) increase hippocampal GR expression in adult rodents. This event leads to decreased hypothalamic Crh expression and an attenuated response upon acute stress exposure over life span (117, 118).

On the other hand, extended periods of maternal separation (up to 4.5 h per day) change the direction of GR expression later in life and manifest with reduced expression of hippocampal GR in rodents. Consequently, negative feedback regulation of the HPA axis is impaired as evidenced by elevated levels of ACTH and corticosterone in adulthood (98). These early life dependent alterations in GR expression can be reversed by housing up-growing animals under an enriched environment which normalizes GR expression, behavioral, and memory performance (119).

## Epigenetic Programing of *GR*

The molecular foundations of epigenetic programing of the *GR* in response to early life care have been discovered by Meaney and Szyf in a series of elegant studies (120) [reviewed in (42, 121)]. Briefly, natural variations in maternal care can stimulate to different degree serotonin turnover in the hippocampus of 6-day-old rat pups, where GR expression is site-specifically increased. Following binding to its receptor, serotonin activates via protein kinase A the expression of the immediate early gene *NGFI-A* encoding a zinc finger transcription factor. NGFI-A protein binds in turn to its recognition site at exon 1_7_ of the proximal *GR* promoter to enhance transcription.

How do these events establish a lasting record of maternal care? Multiple untranslated first exons of the proximal *GR* promoter are transcribed as a result of the associated promoter activity and are spliced on the common second exon to give rise to the same GR protein. The major part of the proximal *GR* promoter, including most alternative exons and the hippocampus-specific exon 1_7_, are contained within a CGI. As alluded above, CGIs can be subject to DNA methylation and serve to control gene expression potential. In this respect the offspring from high-care taking mothers showed decreased CpG methylation at the NGFI-A binding site when compared to offspring of the same age from low-care taking mothers. These differences developed in the first postnatal week concurrent with differences in maternal care and its effect on NGFI-A expression (122). Differences in maternal care disappeared after the first postnatal week and consequently adult rats did not diverge anymore in hippocampal NGF-IA expression. Maintained hypomethylation left, however, an enduring memory trace of the postnatal environment and underpinned increased GR expression restraining HPA-axis activity in response to future stressors.

Epigenetic programing of *GR* occurs also in adult mice. Chronic and acute stress during adulthood induces changes in the methylation level of the GR promoter at exon 1_7_ in the pituitary and adrenal gland whereas brain tissues remained unaffected (123). This result corroborates above findings from *Crh* and suggests that epigenetic programing is tissue-specific dependent on developmental stage.

The hypothesis that early life adversity in humans induces similarly epigenetic programing of hippocampal *GR* expression has been addressed by postmortem brain analysis from suicide victims (124). The documented histories of childhood adversities included severe sexual or physical abuse and neglect. Interestingly, total GR and exon 1F (the human homolog of rat exon 1_7_) expression in the hippocampus was reduced in suicide victims with a history of abuse, but not in those without such a history or in control subjects. These results indicate that *GR* expression associated with early life adversity rather than with circumstances of life leading the way to suicide. These results agree with above findings from rodents that epigenetic programing of GR depends on the developmental context. Together, these studies in human and rat support the concept of a DNA-based memory that underpins the lasting effects of early life adversity.

Does then prenatal adversity also cause epigenetic programing of *GR*? Children whose mothers’ were exposed to enduring stress during pregnancy (i.e., depression, anxiety, death of a relative, or marital discord) suffer from increased rates of psychological and behavioral disorders as adults (103). Hereby, the respective offspring frequently shows signs of HPA-axis deregulation similar to those detected in their depressed mothers (125). In support of above consideration, maternal depression in the third trimester led to an increased neonatal methylation of the presumed NGFI-A DNA-binding site situated at exon 1F in cord blood cells. Additionally, newborn methylation at nucleotide residue CpG3 associated with an enhanced cortisol response to an experimental stressor at 3 months of age (126). These data suggest that gestational depression can elicit epigenetic programing of peripheral and possibly central *GR* expression in neonates which might result in altered HPA-axis responsiveness in small children.

How long does epigenetic programing of neonate *GR* in response to maternal depression last? In a further study exon 1F methylation was measured in a group of children in the range from 10 to 19 years and in their respective mothers. Retrospective psychological evaluations were used to identify those mothers who had been exposed to physical or psychological abuse before, during, or after pregnancy. Exon 1F methylation was increased in adolescent children whose mothers’ had experienced intimate partner violence during pregnancy (127). In contrast, adolescent exon 1F methylation was unaffected in case of prenatal or postnatal maternal abuse pointing to the critical role of gestational stress in epigenetic programing of fetal *GR*. Moreover, none of the respective mothers presented with altered exon 1F methylation in peripheral blood mononuclear cells (PBMC) suggesting that epigenetic programing of *GR* is confined to few sensitive time windows during development.

In further support of this idea, individuals suffering from major depression or borderline personality disorders exhibit increased exon 1F methylation in PBMC. Changes in DNA methylation were more prominent in the latter group and correlated in either with a history of child abuse, its severity, and the exposure to multiple types of maltreatment (128). Interestingly, healthy adults with a history of childhood adversity (maltreatment, poor quality parenting, loss of parents) show as well increased CpG3 methylation at exon 1F in PMBC and an attenuated cortisol response to the dexamethasone/CRH test (129). This finding corroborates that variation in parenting experiences in humans can bring about epigenetic programing of GR and HPA-axis deregulation without clinical manifestation. In this respect, additional stressors appear necessary to give rise to diseases such as depression and PTSD (Figure 5). Collectively, changes in exon 1F methylation seem to fulfill the criteria of an epigenetic risk marker to identify persons at future risk. Follow-up studies are needed to test this hypothesis and to explore exon 1F methylation as a potential biomarker for treatment response and relapse upon disease’s manifestation.

## Gene-Environment Interactions in PTSD

Since the days of Garrod (130), medical research has been puzzled by the fact that the etiology of most common diseases depends not only on discrete genetic and environmental causes but also on the interaction between the two. Separate estimates on the contributions of genes and the environment to a disease without considering their interactions will only incorrectly describe the proportion of the disease (i.e., the population attributable risk) that is explained by genes, the environment, and their joint effects (Figure 5). A landmark discovery in this respect was the finding that the association of a polymorphism in the promoter region of the serotonin transporter gene (SLC6A4, commonly known as 5-HTT) with depression is influenced by stressful life events (131). Following on the concept of gene-environment interaction has been increasingly applied to quantitative epidemiological studies, although the nature of the mechanism(s) underpinning such interplay remained so far elusive. Two recent studies on gene-environment interactions in PTSD shed light on the potential contribution of epigenetic mechanisms as a mediator between risk alleles and early life events.

The neuropeptide PACAP (pituitary adenylate cyclase activating polypeptide) binds to the G-protein coupled receptor PAC1 (132) and is involved in the brain’s response to stress. The PAC1 protein is encoded by the gene *ADCYAP1R1* that has been recently suggested to influence in a sex-specific manner whether an individual will develop PTSD (133). A cohort of urban primary-care patients who had been exposed to severe trauma (child maltreatment, a serious accident or attack with a weapon) showed a positive correlation between peripheral blood levels of PACAP and the extent of posttraumatic stress syndromes, but only in women. High PACAP values correlated with physiological measures of the acoustic startle reflex which has been previously associated with PTSD. Moreover, in a translational animal model the mRNA expression of PAC1 was increased in the amygdala of adult mice following classical fear conditioning and as a function of estrogen exposure.

Notably, a common genetic variant (rs267735) in *ADCYAP1R1* associated with PTSD. This finding was replicated in a second cohort of patients exposed to trauma, once again only in women (133). Differential DNA methylation of *ADCYAP1R1* associated with PTSD symptoms and included the variant rs267735 which maps to a predicted estrogen response element. In support of a regulatory role of this genetic variant, postmortem analysis of female brains evidenced differential expression of PAC1 mRNA as function of the underlying genotype.

Together, these findings offer insight into the question of why women are more likely than men to develop PTSD and suggest that traumatic stress can leave an enduring memory trace on the epigenome by DNA methylation at regulatory polymorphic sites of genes that participate in the stress response. The regulatory site at *ADCYAP1R1* maps to an ERE which undergoes DNA methylation in response to early trauma and thus constitutes an epigenetic risk factor for the later development of PTSD in women.

Similarly, a recent study suggested that a polymorphism in the FK506 binding protein 5 gene (*FKBP5*) can serve as a substrate for early trauma-dependent epigenetic marking to increase the risk for PTSD (134). FKBP5 controls GR function by reducing ligand binding and translocation of the receptor complex to the nucleus (135). Hereby, FKBP5 forms part of a negative, intracellular, ultra-short feedback loop due to GR-dependent transactivation at a glucocorticoid response element (GRE) situated in intron 2. A polymorphism identified in this intron gave rise to a potential TATA element supporting the assembly of the transcriptional machinery. Adults who were homozygous or heterozygous for the A (risk) allele at this polymorphism rather than the G (protective) allele were more likely to suffer from PTSD – provided they had experienced abuse as children. The risk allele conferred an altered chromatin structure and increased transcription of *FKBP5*, hence strengthening the negative feedback loop that attenuated GR activity. Impaired GR function, in turn increases the hormonal response to stress.

How do traumatic experiences then act together with the risk allele in PTSD? Traumatic experiences lead to activation of the GR which is known to initiate changes in DNA methylation (136). Notably, CpG residues in intron 7 of FKBP5 – flanking a predicted GRE – were less methylated in risk allele carriers with a history of childhood abuse compared to those without a history of early trauma or traumatized patients without the risk allele.

In a translational *in vitro* model of early-life stress, treatment of hippocampal progenitor cells with dexamethasone elicited demethylation of intron 7 during proliferation and differentiation. The decline in methylation persisted over 20 days in steroid-free culture compatible with the idea of an enduring epigenetic mark. Furthermore, demethylation in intron 7 of *FKBP5* increased FKBP5 expression, GR resistance, and led to corresponding changes in the expression of downstream genes in PTSD patients harboring the risk allele. In contrast, risk carriers exposed to trauma in adulthood showed maintained DNA methylation at intron 7 suggesting that epigenetic programing of *FKBP5* is confined to a critical time window of hippocampal cell development. Compatible with this view, demethylation at intron 7 in hippocampal neuronal progenitor cells occurred only during the proliferative phase (134).

Consistent with the role of glucocorticoids in controlling Fkbp5 expression via DNA methylation, treatment of adolescent mice with corticosterone across 4 weeks enhanced gene expression in the hippocampus, hypothalamus, and peripheral blood cells (137, 138). This was accompanied by demethylation at intron 5 and 1 in brain tissues and peripheral blood cells, respectively. Interestingly, either intron includes a GRE binding side. Moreover, treatment doses correlated with Fkbp5 expression and demethylation at intron 1 indicating that intron 1 demethylation could serve as a biomarker for prior glucocorticoid load (138).

Together, these findings support the concept that exposure of children carrying the risk allele to stress can elicit enduring epigenetic changes in FKBP5 gene expression predisposing them to stress-associated disorders such as PTSD (Figure 5).

## Conclusion

More than 100 years have passed since Sigmund Freud described the influence of early traumatic experiences on later mental health. Numerous epidemiological, clinical, and translational studies have corroborated the powerful role of early life adversity for the development of depression and PTSD. During sensitive windows of development, adversity can lead to enduring (mal-) programing of the stress system which constitutes an important risk factor for later disease.

Epigenetic mechanisms mediate the dialog between our genes and the environment and can elicit lasting changes in gene expression potential. Experience-dependent epigenetic programing of stress genes is increasingly recognized as an important pathway in the deregulation of stress systems in rodents and patients. Such programing can confer an enhanced risk on disease development upon re-exposure to trauma or stress (Figure 5). Epigenetic programing is, however, not deterministic. In translational models, an enriched environment can attenuate the effects of epigenetic programing. Epigenetic modifications appear amenable across all chapters in one’s life and thus offer great opportunities for timely interventions comprising pharmaceutical and psychotherapeutic treatments.

Nearly twice as much women are affected by stress-related mood disorders compared to men. The HPA axis, the central mediator of the stress response, shows clear sex differences which seem to impact on the prevalence and course of stress-related diseases. Further clinical and translational studies focusing on sex differences in epigenetic programing of HPA-axis activity and brain functions in response to traumatic and social experiences can advance our insight into the sex-bias of PTSD and depression. Epigenetic biomarkers (i.e., *GR* and *FKBP5* in PBMS) in populations at risks appear promising to inform about previous stress exposure and require further clinical investigations.

With the decoding of the human genome at the turn of the millenium, medical research pinned its hope, above all, on the identification of gene variants coming along with an elevated risk of contracting a disease (Figure 5). However, it has become clear that genetic factors and environmental influences are not independent of each other and that acquired pieces of information deliver the instruction how to interpret the genetic blueprint. Whether or not a genetic predisposition manifests as PTSD or depression then seems to depend on how genes and environment conspire. Epigenetic programing in response to trauma or stress can be modified by genetic variation in the underlying substrates. Hereby, silent variations are switched into epigenetic risk factors for PTSD or depression in response to additional adversities (Figure 5). Further studies are needed to address tissue-specificity of experience-dependent programing of genetic variants to distinguish between roles as biomarker, reflecting humoral changes in response to trauma versus roles as epigenetic risk factor driving disease development.

Taken together, epigenetic mechanisms play an important role in experience-dependent programing of stress genes and their genetic variants. Moreover, epigenetic biomarkers encoding previous trauma and stress history might guide early therapeutic interventions and risk management in PTSD and depression on a long-term perspective.

## Conflict of Interest Statement

The authors declare that the research was conducted in the absence of any commercial or financial relationships that could be construed as a potential conflict of interest.

## References

[1] Murray CJ Lopez AD Evidence-based health policy – lessons from the Global Burden of Disease Study. Science (1996) 274:740–3 10.1126/science.274.5288.740 8966556

[2] Wittchen H Jacobi F Rehm J Gustavsson A Svensson M Jönsson B The size and burden of mental disorders and other disorders of the brain in Europe 2010. Eur Neuropsychopharmacol (2011) 21:655–79 10.1016/j.euroneuro.2011.07.018 21896369

[3] Alim TN Graves E Mellman TA Aigbogun N Gray E Lawson W Trauma exposure, posttraumatic stress disorder and depression in an African-American primary care population. J Natl Med Assoc (2006) 98:1630–6 17052054 PMC2569763

[4] Keane TM Marshall AD Taft CT Posttraumatic stress disorder: etiology, epidemiology, and treatment outcome. Annu Rev Clin Psychol (2006) 2:161–97 10.1146/annurev.clinpsy.2.022305.095305 17716068

[5] Cabrera OA Hoge CW Bliese PD Castro CA Messer SC Childhood adversity and combat as predictors of depression and post-traumatic stress in deployed troops. Am J Prev Med (2007) 33:77–82 10.1016/j.amepre.2007.03.019 17673093

[6] Yehuda R McFarlane AC Conflict between current knowledge about posttraumatic stress disorder and its original conceptual basis. Am J Psychiatry (1995) 152:1705–13 8526234 10.1176/ajp.152.12.1705

[7] Hoffmann A Spengler D The lasting legacy of social stress on the epigenome of the hypothalamic-pituitary-adrenal axis. Epigenomics (2012) 4:431–44 10.2217/epi.12.34 22920182

[8] Hoffmann A Spengler D DNA memories of early social life. Neuroscience (2012). 10.1016/j.neuroscience.2012.04.003 22575695

[9] Green JG McLaughlin KA Berglund PA Gruber MJ Sampson NA Zaslavsky AM Childhood adversities and adult psychiatric disorders in the national comorbidity survey replication I: associations with first onset of DSM-IV disorders. Arch Gen Psychiatry (2010) 67:113–23 10.1001/archgenpsychiatry.2009.186 20124111 PMC2822662

[10] Edwards VJ Holden GW Felitti VJ Anda RF Relationship between multiple forms of childhood maltreatment and adult mental health in community respondents: results from the adverse childhood experiences study. Am J Psychiatry (2003) 160:1453–60 10.1176/appi.ajp.160.8.1453 12900308

[11] Felitti VJ Anda RF Nordenberg D Williamson DF Spitz AM Edwards V Relationship of childhood abuse and household dysfunction to many of the leading causes of death in adults. The Adverse Childhood Experiences (ACE) Study. Am J Prev Med (1998) 14:245–58 10.1016/S0749-3797(98)00017-8 9635069

[12] Chapman DP Whitfield CL Felitti VJ Dube SR Edwards VJ Anda RF Adverse childhood experiences and the risk of depressive disorders in adulthood. J Affect Disord (2004) 82:217–25 10.1016/j.jad.2003.12.013 15488250

[13] Dube SR Anda RF Felitti VJ Chapman DP Williamson DF Giles WH Childhood abuse, household dysfunction, and the risk of attempted suicide throughout the life span: findings from the Adverse Childhood Experiences Study. JAMA (2001) 286:3089–96 10.1001/jama.286.24.3089 11754674

[14] Molnar BE Buka SL Kessler RC Child sexual abuse and subsequent psychopathology: results from the National Comorbidity Survey. Am J Public Health (2001) 91:753–60 10.2105/AJPH.91.5.753 11344883 PMC1446666

[15] Levitan RD Rector NA Sheldon T Goering P Childhood adversities associated with major depression and/or anxiety disorders in a community sample of Ontario: issues of co-morbidity and specificity. Depress Anxiety (2003) 17:34–42 10.1002/da.10077 12577276

[16] MacMillan HL Georgiades K Duku EK Shea A Steiner M Niec A Cortisol youths exposed to childhood maltreatment: results of the youth mood project. Biol Psychiatry (2009) 66:62–8 10.1016/j.biopsych.2008.12.014 19217075 PMC3816014

[17] Mullen PE Martin JL Anderson JC Romans SE Herbison GP The long-term impact of the physical, emotional, and sexual abuse of children: a community study. Child Abuse Negl (1996) 20:7–21 10.1016/0145-2134(95)00112-3 8640429

[18] Lang AJ Aarons GA Gearity J Laffaye C Satz L Dresselhaus TR Direct and indirect links between childhood maltreatment, posttraumatic stress disorder, and women’s health. Behav Med (2008) 33:125–35 10.3200/BMED.33.4.125-136 18316270 PMC2547477

[19] Cougle JR Timpano KR Sachs-Ericsson N Keough ME Riccardi CJ Examining the unique relationships between anxiety disorders and childhood physical and sexual abuse in the National Comorbidity Survey-Replication. Psychiatry Res (2010) 177:150–5 10.1016/j.psychres.2009.03.008 20381878

[20] Brewin CR Andrews B Valentine JD Meta-analysis of risk factors for posttraumatic stress disorder in trauma-exposed adults. J Consult Clin Psychol (2000) 68:748–66 10.1037/0022-006X.68.5.748 11068961

[21] Nishith P Mechanic MB Resick PA Prior interpersonal trauma: the contribution to current PTSD symptoms in female rape victims. J Abnorm Psychol (2000) 109:20–5 10.1037/0021-843X.109.1.20 10740932 PMC2962405

[22] Classen CC Sexual revictimization: a review of the empirical literature. Trauma Violence Abuse (2005) 6:103–29 10.1177/1524838005275087 15753196

[23] Margolin G Gordis EB The effects of family and community violence on children. Annu Rev Psychol (2000) 51:445–79 10.1146/annurev.psych.51.1.445 10751978

[24] NCANDS (2011). Child Maltreatment 2011. Available from: http://www.acf.hhs.gov/sites/default/files/cb/cm11.pdf

[25] Sedlak AJ Broadhurst DD (1996). BDD: Executive Summary of the Third National Incidence Study of Child Abuse and Neglect. Available from: https://www.childwelfare.gov/pubs/statsinfo/nis3.cfm

[26] Kessler RC Davis CG Kendler KS Childhood adversity and adult psychiatric disorder in the US National Comorbidity Survey. Psychol Med (1997) 27:1101–19 10.1017/S0033291797005588 9300515

[27] Holmes WC Slap GB Sexual abuse of boys: definition, prevalence, correlates, sequelae, and management. JAMA (1998) 280:1855–62 10.1001/jama.280.21.1855 9846781

[28] NCANDS (2009). Child Maltreatment 2009. Available from: http://archive.acf.hhs.gov/programs/cb/pubs/cm09/cm09.pdf

[29] McLaughlin KA Green JG Gruber MJ Sampson NA Zaslavsky AM Kessler RC Childhood adversities and adult psychiatric disorders in the national comorbidity survey replication II: associations with persistence of DSM-IV disorders. Arch Gen Psychiatry (2010) 67:124–32 10.1001/archgenpsychiatry.2009.187 20124112 PMC2847359

[30] Murgatroyd C Spengler D Epigenetics of early child development. Front Psychiatry (2011) 2:16 10.3389/fpsyt.2011.00016 21647402 PMC3102328

[31] Shonkoff JP Phillips D From Neurons to Neighborhoods. The Science of Early Child Development. Washington, DC: National Academy Press (2000). 25077268

[32] Fox SE Levitt P Nelson CA How the timing and quality of early experiences influence the development of brain architecture. Child Dev (2010) 81:28–40 10.1111/j.1467-8624.2009.01380.x 20331653 PMC2846084

[33] Levine S Infantile experience and resistance to physiological stress. Science (1957) 126:405 10.1126/science.126.3270.405 13467220

[34] Seckl JR Meaney MJ Glucocorticoid programming. Ann N Y Acad Sci (2004) 1032:63–84 10.1196/annals.1314.006 15677396

[35] Kaffman A Meaney MJ Neurodevelopmental sequelae of postnatal maternal care in rodents: clinical and research implications of molecular insights. J Child Psychol Psychiatry (2007) 48:224–44 10.1111/j.1469-7610.2007.01730.x 17355397

[36] Stevens HE Leckman JF Coplan JD Suomi SJ Risk and resilience: early manipulation of macaque social experience and persistent behavioral and neurophysiological outcomes. J Am Acad Child Adolesc Psychiatry (2009) 48:114–27 10.1097/CHI.0b013e318193064c 19127170

[37] Lupien SJ Fiocco A Wan N Maheu F Lord C Schramek T Stress hormones and human memory function across the lifespan. Psychoneuroendocrinology (2005) 30:225–42 10.1016/j.psyneuen.2004.08.003 15511597

[38] Dong M Giles WH Felitti VJ Dube SR Williams JE Chapman DP Insights into causal pathways for ischemic heart disease: adverse childhood experiences study. Circulation (2004) 110:1761–6 10.1161/01.CIR.0000143074.54995.7F 15381652

[39] Anda RF Brown DW Dube SR Bremner JD Felitti VJ Giles WH Adverse childhood experiences and chronic obstructive pulmonary disease in adults. Am J Prev Med (2008) 34:396–403 10.1016/j.amepre.2008.02.002 18407006 PMC8214869

[40] Dube SR Fairweather D Pearson WS Felitti VJ Anda RF Croft JB Cumulative childhood stress and autoimmune diseases in adults. Psychosom Med (2009) 71:243–50 10.1097/PSY.0b013e3181907888 19188532 PMC3318917

[41] Francis DD Caldji C Champagne F Plotsky PM Meaney MJ The role of corticotropin-releasing factor – norepinephrine systems in mediating the effects of early experience on the development of behavioral and endocrine responses to stress. Biol Psychiatry (1999) 46:1153–66 10.1016/S0006-3223(99)00237-1 10560022

[42] Zhang T-Y Bagot R Parent C Nesbitt C Bredy TW Caldji C Maternal programming of defensive responses through sustained effects on gene expression. Biol Psychol (2006) 73:72–89 10.1016/j.biopsycho.2006.01.009 16513241

[43] Andersen SL Teicher MH Stress, sensitive periods and maturational events in adolescent depression. Trends Neurosci (2008) 31:183–91 10.1016/j.tins.2008.01.004 18329735

[44] Kloet ER de Joëls M Holsboer F Stress and the brain: from adaptation to disease. Nat Rev Neurosci (2005) 6:463–75 10.1038/nrn1683 15891777

[45] Gray TS Bingaman EW The amygdala: corticotropin-releasing factor, steroids, and stress. Crit Rev Neurobiol (1996) 10:155–68 10.1615/CritRevNeurobiol.v10.i2.10 8971127

[46] Plotsky PM Cunningham ET Widmaier EP Catecholaminergic modulation of corticotropin-releasing factor and adrenocorticotropin secretion. Endocr Rev (1989) 10:437–58 10.1210/edrv-10-4-437 2558876

[47] Butler PD Weiss JM Stout JC Nemeroff CB Corticotropin-releasing factor produces fear-enhancing and behavioral activating effects following infusion into the locus coeruleus. J Neurosci (1990) 10:176–83 2299391 10.1523/JNEUROSCI.10-01-00176.1990 PMC6570355

[48] Pacak K Palkovits M Kopin IJ Goldstein DS Stress-induced norepinephrine release in the hypothalamic paraventricular nucleus and pituitary-adrenocortical and sympathoadrenal activity: in vivo microdialysis studies. Front Neuroendocrinol (1995) 16:89–150 10.1006/frne.1995.1004 7621982

[49] Nemeroff CB The corticotropin-releasing factor (CRF) hypothesis of depression: new findings and new directions. Mol Psychiatry (1996) 1:336–42 9118360

[50] Cahill L McGaugh JL Mechanisms of emotional arousal and lasting declarative memory. Trends Neurosci (1998) 21:294–9 10.1016/S0166-2236(97)01214-9 9683321

[51] Lee Y Schulkin J Davis M Effect of corticosterone on the enhancement of the acoustic startle reflex by corticotropin releasing factor (CRF). Brain Res (1994) 666:93–8 10.1016/0006-8993(94)90286-0 7889373

[52] Schulkin J Gold PW McEwen BS Induction of corticotropin-releasing hormone gene expression by glucocorticoids: implication for understanding the states of fear and anxiety and allostatic load. Psychoneuroendocrinology (1998) 23:219–43 10.1016/S0306-4530(97)00099-1 9695128

[53] Kraemer GW Ebert MH Schmidt DE McKinney WT A longitudinal study of the effect of different social rearing conditions on cerebrospinal fluid norepinephrine and biogenic amine metabolites in rhesus monkeys. Neuropsychopharmacology (1989) 2:175–89 10.1016/0893-133X(89)90021-3 2477005

[54] Coplan JD Andrews MW Rosenblum LA Owens MJ Friedman S Gorman JM Persistent elevations of cerebrospinal fluid concentrations of corticotropin-releasing factor in adult nonhuman primates exposed to early-life stressors: implications for the pathophysiology of mood and anxiety disorders. Proc Natl Acad Sci U S A (1996) 93:1619–23 10.1073/pnas.93.4.1619 8643680 PMC39991

[55] Holsboer F Ising M Stress hormone regulation: biological role and translation into therapy. Annu Rev Psychol (2010) 61:81–109 10.1146/annurev.psych.093008.100321 19575614

[56] Nemeroff CB Heim CM Thase ME Klein DN Rush AJ Schatzberg AF Differential responses to psychotherapy versus pharmacotherapy in patients with chronic forms of major depression and childhood trauma. Proc Natl Acad Sci U S A (2003) 100:14293–6 10.1073/pnas.2336126100 14615578 PMC283585

[57] Bruce J Fisher PA Pears KC Levine S Morning cortisol Levels in preschool-aged foster children: differential effects of maltreatment type. Dev Psychobiol (2009) 51:14–23 10.1002/dev.20333 18720365 PMC2644049

[58] Goenjian AK Yehuda R Pynoos RS Steinberg AM Tashjian M Yang RK Basal cortisol, dexamethasone suppression of cortisol, and MHPG in adolescents after the 1988 earthquake in Armenia. Am J Psychiatry (1996) 153:929–34 8659616 10.1176/ajp.153.7.929

[59] Trickett PK Noll JG Susman EJ Shenk CE Putnam FW Attenuation of cortisol across development for victims of sexual abuse. Dev Psychopathol (2010) 22:165 10.1017/S0954579409990332 20102654 PMC3100742

[60] Pervanidou P Kolaitis G Charitaki S Lazaropoulou C Papassotiriou I Hindmarsh P The natural history of neuroendocrine changes in pediatric posttraumatic stress disorder (PTSD) after motor vehicle accidents: progressive divergence of noradrenaline and cortisol concentrations over time. Biol Psychiatry (2007) 62:1095–102 10.1016/j.biopsych.2007.02.008 17624319

[61] Resnick HS Yehuda R Pitman RK Foy DW Effect of previous trauma on acute plasma cortisol level following rape. Am J Psychiatry (1995) 152:1675–7 7485635 10.1176/ajp.152.11.1675

[62] McFarlane AC Atchison M Yehuda R The acute stress response following motor vehicle accidents and its relation to PTSD. Ann N Y Acad Sci (1997) 821:437–41 10.1111/j.1749-6632.1997.tb48299.x 9238224

[63] Delahanty DL Raimonde AJ Spoonster E Initial posttraumatic urinary cortisol levels predict subsequent PTSD symptoms in motor vehicle accident victims. Biol Psychiatry (2000) 48:940–7 10.1016/S0006-3223(00)00896-9 11074232

[64] vAllis CD Jenuwein T Reinberg D Epigenetics. Cold Spring Harbor, NY: Cold Spring Harbor Laboratory Press (2007).

[65] Bird A Perceptions of epigenetics. Nature (2007) 447:396–8 10.1038/nature05913 17522671

[66] Goldberg AD Allis CD Bernstein E Epigenetics: a landscape takes shape. Cell (2007) 128:635–8 10.1016/j.cell.2007.02.006 17320500

[67] Rottach A Leonhardt H Spada F DNA methylation-mediated epigenetic control. J Cell Biochem (2009) 108:43–51 10.1002/jcb.22253 19565567

[68] Lehnertz B Ueda Y Derijck AA Braunschweig U Perez-Burgos L Kubicek S Suv39h-mediated histone H3 lysine 9 methylation directs DNA methylation to major satellite repeats at pericentric heterochromatin. Curr Biol (2003) 13:1192–200 10.1016/S0960-9822(03)00432-9 12867029

[69] Viré E Brenner C Deplus R Blanchon L Fraga M Didelot C The Polycomb group protein EZH2 directly controls DNA methylation. Nature (2005) 439:871–4 10.1038/nature04431 16357870

[70] Tachibana M G9a histone methyltransferase plays a dominant role in euchromatic histone H3 lysine 9 methylation and is essential for early embryogenesis. Genes Dev (2002) 16:1779–91 10.1101/gad.989402 12130538 PMC186403

[71] Fuks F Hurd PJ Deplus R Kouzarides T The DNA methyltransferases associate with HP1 and the SUV39H1 histone methyltransferase. Nucleic Acids Res (2003) 31:2305–12 10.1093/nar/gkg332 12711675 PMC154218

[72] Ooi SKT Qiu C Bernstein E Li K Jia D Yang Z DNMT3L connects unmethylated lysine 4 of histone H3 to de novo methylation of DNA. Nature (2007) 448:714–7 10.1038/nature05987 17687327 PMC2650820

[73] Saxonov S Berg P Brutlag DL A genome-wide analysis of CpG dinucleotides in the human genome distinguishes two distinct classes of promoters. Proc Natl Acad Sci U S A (2006) 103:1412–7 10.1073/pnas.0510310103 16432200 PMC1345710

[74] Lister R Pelizzola M Dowen RH Hawkins RD Hon G Tonti-Filippini J Human DNA methylomes at base resolution show widespread epigenomic differences. Nature (2009) 462:315–22 10.1038/nature08514 19829295 PMC2857523

[75] Feng S Cokus SJ Zhang X Chen P-Y Bostick M Goll MG From the cover: conservation and divergence of methylation patterning in plants and animals. Proc Natl Acad Sci U S A (2010) 107:8689–94 10.1073/pnas.1002720107 20395551 PMC2889301

[76] Zemach A McDaniel IE Silva P Zilberman D Genome-wide evolutionary analysis of eukaryotic DNA methylation. Science (2010) 328:916–9 10.1126/science.1186366 20395474

[77] Maunakea AK Nagarajan RP Bilenky M Ballinger TJ D’Souza C Fouse SD Conserved role of intragenic DNA methylation in regulating alternative promoters. Nature (2010) 466:253–7 10.1038/nature09165 20613842 PMC3998662

[78] Wu SC Zhang Y Active DNA demethylation: many roads lead to Rome. Nat Rev Mol Cell Biol (2010) 11:607–20 10.1038/nrm2950 20683471 PMC3711520

[79] Williams K Christensen J Helin K DNA methylation: TET proteins – guardians of CpG islands? EMBO Rep (2011) 13:28–35 10.1038/embor.2011.233 22157888 PMC3246258

[80] Guo JU Su Y Zhong C Ming G-L Song H Emerging roles of TET proteins and 5-hydroxymethylcytosines in active DNA demethylation and beyond. Cell Cycle (2011) 10:2662–8 10.4161/cc.10.16.17093 21811096 PMC3219536

[81] Spruijt CG Gnerlich F Smits AH Pfaffeneder T Jansen PW Bauer C Dynamic readers for 5-(hydroxy)methylcytosine and its oxidized derivatives. Cell (2013) 152:1146–59 10.1016/j.cell.2013.02.004 23434322

[82] Yildirim O Li R Hung J-H Chen PB Dong X Ee L-S Mbd3/NURD complex regulates expression of 5-hydroxymethylcytosine marked genes in embryonic stem cells. Cell (2011) 147:1498–510 10.1016/j.cell.2011.11.054 22196727 PMC3252821

[83] Griffith JS Mahler HR DNA ticketing theory of memory. Nature (1969) 223:580–2 10.1038/223580a0 5799529

[84] Holliday R Is there an epigenetic component in long-term memory? J Theor Biol (1999) 200:339–41 10.1006/jtbi.1999.0995 10527722

[85] Bird A DNA methylation patterns and epigenetic memory. Genes Dev (2002) 16:6–21 10.1101/gad.947102 11782440

[86] Murgatroyd C Wu Y Bockmühl Y Spengler D Genes learn from stress: how infantile trauma programs us for depression. Epigenetics (2010) 5:194–9 10.4161/epi.5.3.11375 375 20339319

[87] Avishai-Eliner S Yi SJ Newth CJ Baram TZ Effects of maternal and sibling deprivation on basal and stress induced hypothalamic-pituitary-adrenal components in the infant rat. Neurosci Lett (1995) 192:49–52 10.1016/0304-3940(95)11606-W 7675308 PMC3498456

[88] Smith MA Kim SY van Oers HJ Levine S Maternal deprivation and stress induce immediate early genes in the infant rat brain. Endocrinology (1997) 138:4622–8 10.1210/en.138.11.4622 9348187

[89] Schmidt M Enthoven L van Woezik JHG Levine S de Kloet ER Oitzl MS The dynamics of the hypothalamic-pituitary-adrenal axis during maternal deprivation. J Neuroendocrinol (2004) 16:52–7 10.1111/j.1365-2826.2004.01123.x 14962076

[90] Enthoven L Oitzl MS Koning N van der Mark M de Kloet ER Hypothalamic-pituitary-adrenal axis activity of newborn mice rapidly desensitizes to repeated maternal absence but becomes highly responsive to novelty. Endocrinology (2008) 149:6366–77 10.1210/en.2008-0238 18635659

[91] Daskalakis NP Claessens SE Laboyrie JJ Enthoven L Oitzl MS Champagne DL The newborn rat’s stress system readily habituates to repeated and prolonged maternal separation, while continuing to respond to stressors in context dependent fashion. Horm Behav (2011) 60:165–76 10.1016/j.yhbeh.2011.04.003 21570400

[92] Baram TZ Hatalski CG Neuropeptide-mediated excitability: a key triggering mechanism for seizure generation in the developing brain. Trends Neurosci (1998) 21:471–6 10.1016/S0166-2236(98)01275-2 9829688 PMC3372323

[93] van Oers HJ de Kloet ER Whelan T Levine S Maternal deprivation effect on the infant’s neural stress markers is reversed by tactile stimulation and feeding but not by suppressing corticosterone. J Neurosci (1998) 18:10171–9 9822770 10.1523/JNEUROSCI.18-23-10171.1998 PMC6793306

[94] Dent GW Smith MA Levine S Rapid induction of corticotropin-releasing hormone gene transcription in the paraventricular nucleus of the developing rat. Endocrinology (2000) 141:1593–8 10.1210/en.141.5.1593 10803566

[95] Liu D Maternal care, hippocampal glucocorticoid receptors, and hypothalamic-pituitary-adrenal responses to stress. Science (1997) 277:1659–62 10.1126/science.277.5332.1659 9287218

[96] Caldji C Tannenbaum B Sharma S Francis D Plotsky PM Meaney MJ Maternal care during infancy regulates the development of neural systems mediating the expression of fearfulness in the rat. Proc Natl Acad Sci U S A (1998) 95:5335–40 10.1073/pnas.95.9.5335 9560276 PMC20261

[97] Plotsky PM Thrivikraman KV Nemeroff CB Caldji C Sharma S Meaney MJ Long-term consequences of neonatal rearing on central corticotropin-releasing factor systems in adult male rat offspring. Neuropsychopharmacology (2005) 30:2192–204 10.1038/sj.npp.1300769 15920504

[98] Plotsky PM Meaney MJ Early, postnatal experience alters hypothalamic corticotropin-releasing factor (CRF) mRNA, median eminence CRF content and stress-induced release in adult rats. Brain Res Mol Brain Res (1993) 18:195–200 10.1016/0169-328X(93)90189-V 8497182

[99] Avishai-Eliner S Eghbal-Ahmadi M Tabachnik E Brunson KL Baram TZ Down-regulation of hypothalamic corticotropin-releasing hormone messenger ribonucleic acid (mRNA) precedes early-life experience-induced changes in hippocampal glucocorticoid receptor mRNA. Endocrinology (2001) 142:89–97 10.1210/en.142.1.89 11145570 PMC3100725

[100] Fenoglio KA Neuroplasticity of the hypothalamic-pituitary-adrenal axis early in life requires recurrent recruitment of stress-regulating brain regions. J Neurosci (2006) 26:2434–42 10.1523/JNEUROSCI.4080-05.2006 16510721 PMC2408688

[101] Gilles EE Schultz L Baram TZ Abnormal corticosterone regulation in an immature rat model of continuous chronic stress. Pediatr Neurol (1996) 15:114–9 10.1016/0887-8994(96)00153-1 8888044 PMC3415889

[102] Avishai-Eliner S Gilles EE Eghbal-Ahmadi M Bar-El Y Baram TZ Altered regulation of gene and protein expression of hypothalamic-pituitary-adrenal axis components in an immature rat model of chronic stress. J Neuroendocrinol (2001) 13:799–807 10.1046/j.1365-2826.2001.00698.x 11578530 PMC3100736

[103] Talge NM Neal C Glover V Antenatal maternal stress and long-term effects on child neurodevelopment: how and why? J Child Psychol Psychiatry (2007) 48:245–61 10.1111/j.1469-7610.2006.01714.x 17355398 PMC11016282

[104] Mueller BR Bale TL Sex-specific programming of offspring emotionality after stress early in pregnancy. J Neurosci (2008) 28:9055–65 10.1523/JNEUROSCI.1424-08.2008 18768700 PMC2731562

[105] Chen J Evans AN Liu Y Honda M Saavedra JM Aguilera G Maternal deprivation in rats is associated with corticotrophin-releasing hormone (CRH) promoter hypomethylation and enhances CRH transcriptional responses to stress in adulthood. J Neuroendocrinol (2012) 24:1055–64 10.1111/j.1365-2826.2012.02306.x 22375940 PMC3380160

[106] Elliott E Ezra-Nevo G Regev L Neufeld-Cohen A Chen A Resilience to social stress coincides with functional DNA methylation of the Crf gene in adult mice. Nat Neurosci (2010) 13:1351–3 10.1038/nn.2642 20890295

[107] Ma DK Jang M-H Guo JU Kitabatake Y Chang M-L Powanpongkul N Neuronal activity-induced Gadd45b promotes epigenetic DNA demethylation and adult neurogenesis. Science (2009) 323:1074–7 10.1126/science.1166859 19119186 PMC2726986

[108] Schafer A Karaulanov E Stapf U Doderlein G Niehrs C Ing1 functions in DNA demethylation by directing Gadd45a to H3K4me3. Genes Dev (2013) 27:261–73 10.1101/gad.186916.112 23388825 PMC3576512

[109] Sterrenburg L Gaszner B Boerrigter J Santbergen L Bramini M Elliott E Chronic stress induces sex-specific alterations in methylation and expression of corticotropin-releasing factor gene in the rat. PLoS ONE (2011) 6:e28128 10.1371/journal.pone.0028128 22132228 PMC3223222

[110] Menger Y Bettscheider M Murgatroyd C Spengler D Sex differences in brain epigenetics. Epigenomics (2010) 2:807–21 10.2217/epi.10.60 22122084

[111] Todeschin AS Winkelmann-Duarte EC Jacob MHV Aranda BCC Jacobs S Fernandes MC Effects of neonatal handling on social memory, social interaction, and number of oxytocin and vasopressin neurons in rats. Horm Behav (2009) 56:93–100 10.1016/j.yhbeh.2009.03.006 19324045

[112] Veenema AH Bredewold R Neumann ID Opposite effects of maternal separation on intermale and maternal aggression in C57BL/6 mice: link to hypothalamic vasopressin and oxytocin immunoreactivity. Psychoneuroendocrinology (2007) 32:437–50 10.1016/j.psyneuen.2007.02.008 17433558

[113] Francis DD Young LJ Meaney MJ Insel TR Naturally occurring differences in maternal care are associated with the expression of oxytocin and vasopressin (V1a) receptors: gender differences. J Neuroendocrinol (2002) 14:349–53 10.1046/j.0007-1331.2002.00776.x 12000539

[114] Marshall AD Posttraumatic stress disorder and partner-specific social cognition: a pilot study of sex differences in the impact of arginine vasopressin. Biol Psychol (2013) 93:296–303 10.1016/j.biopsycho.2013.02.014 23470513 PMC3644358

[115] Murgatroyd C Patchev AV Wu Y Micale V Bockmühl Y Fischer D Dynamic DNA methylation programs persistent adverse effects of early-life stress. Nat Neurosci (2009) 12:1559–66 10.1038/nn.2436 19898468

[116] Guy J Cheval H Selfridge J Bird A The role of MeCP2 in the brain. Annu Rev Cell Dev Biol (2011) 27:631–52 10.1146/annurev-cellbio-092910-154121 21721946

[117] Meaney MJ Aitken DH Viau V Sharma S Sarrieau A Neonatal handling alters adrenocortical negative feedback sensitivity and hippocampal type II glucocorticoid receptor binding in the rat. Neuroendocrinology (1989) 50:597–604 10.1159/000125287 2558328

[118] Meaney MJ Aitken DH Sharma S Viau V Basal ACTH, corticosterone and corticosterone-binding globulin levels over the diurnal cycle, and age-related changes in hippocampal type I and type II corticosteroid receptor binding capacity in young and aged, handled and nonhandled rats. Neuroendocrinology (1992) 55:204–13 10.1159/000126116 1320217

[119] Francis DD Diorio J Plotsky PM Meaney MJ Environmental enrichment reverses the effects of maternal separation on stress reactivity. J Neurosci (2002) 22:7840–3 12223535 10.1523/JNEUROSCI.22-18-07840.2002 PMC6758090

[120] Meaney MJ Szyf M Maternal care as a model for experience-dependent chromatin plasticity? Trends Neurosci (2005) 28:456–63 10.1016/j.tins.2005.07.006 16054244

[121] Zhang T-Y Meaney MJ Epigenetics and the environmental regulation of the genome and its function. Annu Rev Psychol (2010) 61:439–66 10.1146/annurev.psych.60.110707.163625 19958180

[122] Weaver ICG Cervoni N Champagne FA D’Alessio AC Sharma S Seckl JR Epigenetic programming by maternal behavior. Nat Neurosci (2004) 7:847–54 10.1038/nn1276 15220929

[123] Witzmann SR Turner JD Mériaux SB Meijer OC Muller CP Epigenetic regulation of the glucocorticoid receptor promoter 1(7) in adult rats. Epigenetics (2012) 7:1290–301 10.4161/epi.22363 23023726 PMC3499330

[124] McGowan PO Sasaki A D’Alessio AC Dymov S Labonté B, Szyf M Epigenetic regulation of the glucocorticoid receptor in human brain associates with childhood abuse. Nat Neurosci (2009) 12:342–8 10.1038/nn.2270 19234457 PMC2944040

[125] Markham JA Koenig JI Prenatal stress: role in psychotic and depressive diseases. Psychopharmacology (Berl) (2011) 214:89–106 10.1007/s00213-010-2035-0 20949351 PMC3050113

[126] Oberlander TF Weinberg J Papsdorf M Grunau R Misri S Devlin AM Prenatal exposure to maternal depression, neonatal methylation of human glucocorticoid receptor gene (NR3C1) and infant cortisol stress responses. Epigenetics (2008) 3:97–106 10.4161/epi.3.2.6034 18536531

[127] Radtke KM Ruf M Gunter HM Dohrmann K Schauer M Meyer A Transgenerational impact of intimate partner violence on methylation in the promoter of the glucocorticoid receptor. Transl Psychiatry (2011) 1:e21 10.1038/tp.2011.21 22832523 PMC3309516

[128] Perroud N Paoloni-Giacobino A Prada P Olié E, Salzmann A Nicastro R Increased methylation of glucocorticoid receptor gene (NR3C1) in adults with a history of childhood maltreatment: a link with the severity and type of trauma. Transl Psychiatry (2011) 1:e59 10.1038/tp.2011.60 22832351 PMC3309499

[129] Tyrka AR Price LH Marsit C Walters OC Carpenter LL Uddin M Childhood adversity and epigenetic modulation of the leukocyte glucocorticoid receptor: preliminary findings in healthy adults. PLoS ONE (2012) 7:e30148 10.1371/journal.pone.0030148 22295073 PMC3266256

[130] Garrod AE About alkaptonuria. Med Chir Trans (1902) 85:69–78 20896987 PMC2036375

[131] Caspi A Sugden K Moffitt TE Taylor A Craig IW Harrington H Influence of life stress on depression: moderation by a polymorphism in the 5-HTT gene. Science (2003) 301:386–9 10.1126/science.1083968 12869766

[132] Spengler D Waeber C Pantaloni C Holsboer F Bockaert J Seeburg PH Differential signal transduction by five splice variants of the PACAP receptor. Nature (1993) 365:170–5 10.1038/365170a0 8396727

[133] Ressler KJ Mercer KB Bradley B Jovanovic T Mahan A Kerley K Post-traumatic stress disorder is associated with PACAP and the PAC1 receptor. Nature (2011) 470:492–7 10.1038/nature09856 21350482 PMC3046811

[134] Klengel T Mehta D Anacker C Rex-Haffner M Pruessner JC Pariante CM Allele-specific FKBP5 DNA demethylation mediates gene – childhood trauma interactions. Nat Neurosci (2012) 16:33–41 10.1038/nn.3275 23201972 PMC4136922

[135] Wochnik GM Rüegg J Abel GA Schmidt U Holsboer F Rein T FK506-binding proteins 51 and 52 differentially regulate dynein interaction and nuclear translocation of the glucocorticoid receptor in mammalian cells. J Biol Chem (2005) 280:4609–16 10.1074/jbc.M407498200 15591061

[136] Grange T Cappabianca L Flavin M Sassi H Thomassin H In vivo analysis of the model tyrosine aminotransferase gene reveals multiple sequential steps in glucocorticoid receptor action. Oncogene (2001) 20:3028–38 10.1038/sj.onc.1204327 11420718

[137] Lee RS Tamashiro KLK Yang X Purcell RH Harvey A Willour VL Chronic corticosterone exposure increases expression and decreases deoxyribonucleic acid methylation of Fkbp5 in mice. Endocrinology (2010) 151:4332–43 10.1210/en.2010-0225 20668026 PMC2940504

[138] Lee RS Tamashiro KLK Yang X Purcell RH Huo Y Rongione M A measure of glucocorticoid load provided by DNA methylation of Fkbp5 in mice. Psychopharmacology (Berl) (2011) 218:303–12 10.1007/s00213-011-2307-3 21509501 PMC3918452

[139] Nemeroff CB Widerlöv E Bissette G Walléus H Karlsson I Eklund K Elevated concentrations of CSF corticotropin-releasing factor-like immunoreactivity in depressed patients. Science (1984) 226:1342–4 10.1126/science.6334362 6334362

[140] Carpenter LL Tyrka AR McDougle CJ Malison RT Owens MJ Nemeroff CB Cerebrospinal fluid corticotropin-releasing factor and perceived early-life stress in depressed patients and healthy control subjects. Neuropsychopharmacology (2004) 29:777–84 10.1038/sj.npp.1300375 14702025

[141] Raadsheer FC Hoogendijk WJ Stam FC Tilders FJ Swaab DF Increased numbers of corticotropin-releasing hormone expressing neurons in the hypothalamic paraventricular nucleus of depressed patients. Neuroendocrinology (1994) 60:436–44 10.1159/000126778 7824085

[142] Wang S-S Kamphuis W Huitinga I Zhou J-N Swaab DF Gene expression analysis in the human hypothalamus in depression by laser microdissection and real-time PCR: the presence of multiple receptor imbalances. Mol Psychiatry (2008) 13(786–99):741 10.1038/mp.2008.38 18427561

[143] Sachar EJ Roffwarg HP Gruen PH Altman N Sassin J Neuroendocrine studies of depressive illness. Pharmakopsychiatr Neuropsychopharmakol (1976) 9:11–7 10.1055/s-0028-1094472 981319

[144] Shea AK Streiner DL Fleming A Kamath MV Broad K Steiner M The effect of depression, anxiety and early life trauma on the cortisol awakening response during pregnancy: preliminary results. Psychoneuroendocrinology (2007) 32:1013–20 10.1016/j.psyneuen.2007.07.006 17855000

[145] Holsboer F Liebl R Hofschuster E Repeated dexamethasone suppression test during depressive illness. Normalisation of test result compared with clinical improvement. J Affect Disord (1982) 4:93–101 10.1016/0165-0327(82)90039-8 6213695

[146] Heim C Newport DJ Bonsall R Miller AH Nemeroff CB Altered pituitary-adrenal axis responses to provocative challenge tests in adult survivors of childhood abuse. Am J Psychiatry (2001) 158:575–81 10.1176/appi.ajp.158.4.575 11282691

[147] Holsboer F Gerken A Bardeleben U von Grimm W Beyer H Müller OA Human corticotropin-releasing hormone in depression – correlation with thyrotropin secretion following thyrotropin-releasing hormone. Biol Psychiatry (1986) 21:601–11 10.1016/0006-3223(86)90121-6 3011129

[148] Gerra G Leonardi C Cortese E Zaimovic A Dell’Agnello G Manfredini M Adrenocorticotropic hormone and cortisol plasma levels directly correlate with childhood neglect and depression measures in addicted patients. Addict Biol (2008) 13:95–104 10.1111/j.1369-1600.2007.00086.x 18201294

[149] Wingenfeld K Driessen M Adam B Hill A Overnight urinary cortisol release in women with borderline personality disorder depends on comorbid PTSD and depressive psychopathology. Eur Psychiatry (2007) 22:309–12 10.1016/j.eurpsy.2006.09.002 17142011

[150] Heim C Mletzko T Purselle D Musselman DL Nemeroff CB The dexamethasone/corticotropin-releasing factor test in men with major depression: role of childhood trauma. Biol Psychiatry (2008) 63:398–405 10.1016/j.biopsych.2007.07.002 17825799

[151] Carvalho Fernando S Beblo T Schlosser N Terfehr K Otte C Löwe B Associations of childhood trauma with hypothalamic-pituitary-adrenal function in borderline personality disorder and major depression. Psychoneuroendocrinology (2012) 37:1659–68 10.1016/j.psyneuen.2012.02.012 22444624

[152] Power C Thomas C Li L Hertzman C Childhood psychosocial adversity and adult cortisol patterns. Br J Psychiatry (2012) 201:199–206 10.1192/bjp.bp.111.096032 22790680

[153] Hinkelmann K Moritz S Botzenhardt J Muhtz C Wiedemann K Kellner M Changes in cortisol secretion during antidepressive treatment and cognitive improvement in patients with major depression: a longitudinal study. Psychoneuroendocrinology (2012) 37:685–92 10.1016/j.psyneuen.2011.08.012 21944955

[154] Messerli-Bürgy N Molloy GJ Wikman A Perkins-Porras L Randall G Steptoe A Cortisol levels and history of depression in acute coronary syndrome patients. Psychol Med (2012) 42:1815–23 10.1017/S0033291711002959 22234288

[155] Yilmaz Z Kaplan AS Levitan RD The role of depression and childhood trauma on cortisol suppression in women with bulimia nervosa: a pilot study. Eat Weight Disord (2012) 17:e17–21 22751268 10.1007/BF03325324

[156] Carroll BJ Curtis GC Mendels J Neuroendocrine regulation in depression. I. Limbic system-adrenocortical dysfunction. Arch Gen Psychiatry (1976) 33:1039–44 10.1001/archpsyc.1976.01770090029002 962488

[157] Newport D Heim C Bonsall R Miller AH Nemeroff CB Pituitary-adrenal responses to standard and low-dose dexamethasone suppression tests in adult survivors of child abuse. Biol Psychiatry (2004) 55:10–20 10.1016/S0006-3223(03)00692-9 14706420

[158] Modell S Yassouridis A Huber J Holsboer F Corticosteroid receptor function is decreased in depressed patients. Neuroendocrinology (1997) 65:216–22 10.1159/000127275 9088003

[159] Bremner JD Licinio J Darnell A Krystal JH Owens MJ Southwick SM Elevated CSF corticotropin-releasing factor concentrations in posttraumatic stress disorder. Am J Psychiatry (1997) 154:624–9 9137116 10.1176/ajp.154.5.624 PMC3233756

[160] Lee RJ Gollan J Kasckow J Geracioti T Coccaro EF CSF corticotropin-releasing factor in personality disorder: relationship with self-reported parental care. Neuropsychopharmacology (2006) 31:2289–95 16880775 10.1038/sj.npp.1301104

[161] Baker DG West SA Nicholson WE Ekhator NN Kasckow JW Hill KK Serial CSF corticotropin-releasing hormone levels and adrenocortical activity in combat veterans with posttraumatic stress disorder. Am J Psychiatry (1999) 156:585–8 10200738 10.1176/ajp.156.4.585

[162] de Kloet CS Vermetten E Geuze E Lentjes EGWM Heijnen CJ Stalla GK Elevated plasma corticotrophin-releasing hormone levels in veterans with posttraumatic stress disorder. Prog Brain Res (2008) 167:287–91 10.1016/S0079-6123(07)67025-3 18037027

[163] Carlson M Earls F Psychological and neuroendocrinological sequelae of early social deprivation in institutionalized children in Romania. Ann N Y Acad Sci (1997) 807:419–28 10.1111/j.1749-6632.1997.tb51936.x 9071367

[164] Bremner D Vermetten E Kelley ME Cortisol, dehydroepiandrosterone, and estradiol measured over 24 hours in women with childhood sexual abuse-related posttraumatic stress disorder. J Nerv Ment Dis (2007) 195:919–27 10.1097/NMD.0b013e3181594ca0 18000454

[165] King JA Mandansky D King S Fletcher KE Brewer J Early sexual abuse and low cortisol. Psychiatry Clin Neurosci (2001) 55:71–4 10.1046/j.1440-1819.2001.00787.x 11235861

[166] Brand SR Brennan PA Newport DJ Smith AK Weiss T Stowe ZN The impact of maternal childhood abuse on maternal and infant HPA axis function in the postpartum period. Psychoneuroendocrinology (2010) 35:686–93 10.1016/j.psyneuen.2009.10.009 19931984 PMC2862800

[167] Kliewer W Violence exposure and cortisol responses in urban youth. Int J Behav Med (2006) 13:109–20 10.1207/s15327558ijbm1302_2 16712428

[168] Yehuda R Southwick SM Nussbaum G Wahby V Giller EL Mason JW Low urinary cortisol excretion in patients with posttraumatic stress disorder. J Nerv Ment Dis (1990) 178:366–9 10.1097/00005053-199006000-00004 2348190

[169] Bevans K Cerbone A Overstreet S Relations between recurrent trauma exposure and recent life stress and salivary cortisol among children. Dev Psychopathol (2008) 20:257–72 10.1017/S0954579408000126 18211737

[170] Boscarino JA Posttraumatic stress disorder, exposure to combat, and lower plasma cortisol among Vietnam veterans: findings and clinical implications. J Consult Clin Psychol (1996) 64:191–201 10.1037/0022-006X.64.1.191 8907099

[171] Badanes LS Watamura SE Hankin BL Hypocortisolism as a potential marker of allostatic load in children: associations with family risk and internalizing disorders. Dev Psychopathol (2011) 23:881–96 10.1017/S095457941100037X 21756439 PMC4072203

[172] Yehuda R Teicher MH Trestman RL Levengood RA Siever LJ Cortisol regulation in posttraumatic stress disorder and major depression: a chronobiological analysis. Biol Psychiatry (1996) 40:79–88 10.1016/0006-3223(95)00451-3 8793040

[173] Hauser S Reck C Möller M Resch F Maser-Gluth C Möhler E Kindliches temperament und müterliche affektivität. Prax Kinderpsychol Kinderpsychiatr (2012) 61:92–107 22462293 10.13109/prkk.2012.61.2.92

[174] Thaller V Vrkljan M Hotujac L Thakore J The potential role of hypocortisolism in the pathophysiology of PTSD and psoriasis. Coll Antropol (1999) 23:611–9 10646236

[175] Rohleder N Joksimovic L Wolf JM Kirschbaum C Hypocortisolism and increased glucocorticoid sensitivity of pro-Inflammatory cytokine production in Bosnian war refugees with posttraumatic stress disorder. Biol Psychiatry (2004) 55:745–51 10.1016/j.biopsych.2003.11.018 15039004

[176] Bierer LM Tischler L Labinsky E Cahill S Foa E Yehuda R Clinical correlates of 24-h cortisol and norepinephrine excretion among subjects seeking treatment following the world trade center attacks on 9/11. Ann N Y Acad Sci (2006) 1071:514–20 10.1196/annals.1364.055 16891610

[177] Brand SR Engel SM Canfield RL Yehuda R The effect of maternal PTSD following in utero trauma exposure on behavior and temperament in the 9-month-old infant. Ann N Y Acad Sci (2006) 1071:454–8 10.1196/annals.1364.041 16891597

[178] Brewer-Smyth K Burgess AW Childhood sexual abuse by a family member, salivary cortisol, and homicidal behavior of female prison inmates. Nurs Res (2008) 57:166–74 10.1097/01.NNR.0000319501.97864.d5 18496102

[179] Flory JD Yehuda R Grossman R New AS Mitropoulou V Siever LJ Childhood trauma and basal cortisol in people with personality disorders. Compr Psychiatry (2009) 50:34–7 10.1016/j.comppsych.2008.05.007 19059511 PMC2614618

[180] Weissbecker I Floyd A Dedert E Salmon P Sephton S Childhood trauma and diurnal cortisol disruption in fibromyalgia syndrome. Psychoneuroendocrinology (2006) 31:312–24 10.1016/j.psyneuen.2005.08.009 16274933

[181] van der Vegt EJ van der Ende J Kirschbaum C Verhulst FC Tiemeier H Early neglect and abuse predict diurnal cortisol patterns in adults. Psychoneuroendocrinology (2009) 34:660–9 10.1016/j.psyneuen.2008.11.004 19128884

[182] Yehuda R Lowy MT Southwick SM Shaffer D Giller EL Lymphocyte glucocorticoid receptor number in posttraumatic stress disorder. Am J Psychiatry (1991) 148:499–504 2006697 10.1176/ajp.148.4.499

[183] Yehuda R Boisoneau D Lowy MT Giller EL Dose-response changes in plasma cortisol and lymphocyte glucocorticoid receptors following dexamethasone administration in combat veterans with and without posttraumatic stress disorder. Arch Gen Psychiatry (1995) 52:583–93 10.1001/archpsyc.1995.03950190065010 7598635

[184] Yehuda R Cai G Golier JA Sarapas C Galea S Ising M Gene expression patterns associated with posttraumatic stress disorder following exposure to the world trade center attacks. Biol Psychiatry (2009) 66:708–11 10.1016/j.biopsych.2009.02.034 19393990

[185] Duval F Crocq M-A Guillon M-S Mokrani M-C Monreal J Bailey P Increased adrenocorticotropin suppression after dexamethasone administration in sexually abused adolescents with posttraumatic stress disorder. Ann N Y Acad Sci (2004) 1032:273–5 10.1196/annals.1314.036 15677426

[186] Stein MB Yehuda R Koverola C Hanna C Enhanced dexamethasone suppression of plasma cortisol in adult women traumatized by childhood sexual abuse. Biol Psychiatry (1997) 42:680–6 10.1016/S0006-3223(96)00489-1 9325561

[187] Yehuda R Golier JA Halligan SL Meaney M Bierer LM The ACTH response to dexamethasone in PTSD. Am J Psychiatry (2004) 161:1397–403 10.1176/appi.ajp.161.8.1397 15285965

[188] Heim C Ehlert U Hanker JP Hellhammer DH Abuse-related posttraumatic stress disorder and alterations of the hypothalamic-pituitary-adrenal axis in women with chronic pelvic pain. Psychosom Med (1998) 60:309–18 9625218 10.1097/00006842-199805000-00017

[189] Ströhle A Scheel M Modell S Holsboer F Blunted ACTH response to dexamethasone suppression-CRH stimulation in posttraumatic stress disorder. J Psychiatr Res (2008) 42:1185–8 10.1016/j.jpsychires.2008.01.015 18342888

[190] Carpenter LL Tyrka AR Ross NS Khoury L Anderson GM Price LH Effect of childhood emotional abuse and age on cortisol responsivity in adulthood. Biol Psychiatry (2009) 66:69–75 10.1016/j.biopsych.2009.02.030 19375070 PMC2696583

